# Enhancing *E. coli* Tolerance towards Oxidative Stress via Engineering Its Global Regulator cAMP Receptor Protein (CRP)

**DOI:** 10.1371/journal.pone.0051179

**Published:** 2012-12-14

**Authors:** Souvik Basak, Rongrong Jiang

**Affiliations:** School of Chemical & Biomedical Engineering, Nanyang Technological University, Singapore, Singapore; University of California Merced, United States of America

## Abstract

Oxidative damage to microbial hosts often occurs under stressful conditions during bioprocessing. Classical strain engineering approaches are usually both time-consuming and labor intensive. Here, we aim to improve *E. coli* performance under oxidative stress *via* engineering its global regulator cAMP receptor protein (CRP), which can directly or indirectly regulate redox-sensing regulators SoxR and OxyR, and other ∼400 genes in *E. coli*. Error-prone PCR technique was employed to introduce modifications to CRP, and three mutants (OM1∼OM3) were identified with improved tolerance *via* H_2_O_2_ enrichment selection. The best mutant OM3 could grow in 12 mM H_2_O_2_ with the growth rate of 0.6 h^−1^, whereas the growth of wild type was completely inhibited at this H_2_O_2_ concentration. OM3 also elicited enhanced thermotolerance at 48°C as well as resistance against cumene hydroperoxide. The investigation about intracellular reactive oxygen species (ROS), which determines cell viability, indicated that the accumulation of ROS in OM3 was always lower than in WT with or without H_2_O_2_ treatment. Genome-wide DNA microarray analysis has shown not only CRP-regulated genes have demonstrated great transcriptional level changes (up to 8.9-fold), but also RpoS- and OxyR-regulated genes (up to 7.7-fold). qRT-PCR data and enzyme activity assay suggested that catalase (*katE*) could be a major antioxidant enzyme in OM3 instead of alkyl hydroperoxide reductase or superoxide dismutase. To our knowledge, this is the first work on improving *E. coli* oxidative stress resistance by reframing its transcription machinery through its native global regulator. The positive outcome of this approach may suggest that engineering CRP can be successfully implemented as an efficient strain engineering alternative for *E. coli*.

## Introduction

Strain engineering approaches have been widely implemented for the production of a broad range of compounds [1], [2], [3], [4], [5], [6], [7]. Using UV/chemical mutagens or rewiring metabolic pathways through gene addition/knockout have been traditional approaches for strain improvement [8]. Classical strain engineering strategies are often time-consuming and labor-intensive [9]. The manipulation of metabolic pathways, however, needs comprehensive knowledge of the complex metabolic network together with the fitness of the manipulation in the phenotypic context [10]. Moreover, a functional cluster of genes orchestrate phenotypic modulation only when perturbed altogether [11], which is difficult to be achieved by metabolic approach.

The strategy of reprogramming a network of genes for phenotype enhancement has led to transcriptional engineering that enables reframing genetic control circuits by modification to entire genomic hierarchy inside microorganisms [12]. Global regulators are able to organize a large repertoire of genetic switches [13]. These regulators can also impart pleiotropic phenotype changes through the regulation of operons belonging to various functional groups [14]. Transcriptional engineering has been evolved as a potential tool for strain engineering over the last few years to alter strain stress tolerance [15], [16], [17], biofuel production [18], [19], [20], and biofilm formation [21], [22].

In this work, we focus on engineering global regulator cAMP receptor protein (CRP) of *E. coli* to improve its performance under stress. Seven global regulators (ArcA, CRP, FIS, FNR, IHF, LRP and H-NS) in *E. coli* can regulate about half of the total genes [23]. Among them, CRP can regulate more than 400 genes and harmonize certain genetic circuits by directly or indirectly regulating other transcriptional regulators [24], which makes it a potential target for altering cellular phenotypes. Previously, we have shown that engineering CRP can improve *E. coli* osmotolerance [25], 1-butanol tolerance [26] and organic solvent tolerance [27]. Here, we aim to explore the possibility of rewiring CRP against oxidative damage often encountered inside bioreactors under stressful conditions [28]. *E. coli* DH5α was used as host strain for its suitability in plasmid stability and in bioprocess usage [29], [30].

Oxidative modification of biological macromolecules and intracellular components by reactive oxygen species (ROS) such as superoxide anion (O_2_ **^.^** ^−^), hydrogen peroxide (H_2_O_2_), and hydroxyl radical (OH**^.^**), can lead to cell damage [31]. The prototype response of *E. coli* against oxidative stress is the induction of antioxidant enzymes involved in ROS scavenging and DNA repair [32], which is *via* global transcriptional activation of redox-sensing regulators SoxR and OxyR [33]. In addition, oxidative stress in *E. coli* also induces chaperone such as Hsp33 to protect plenty of cellular proteins from stress generated shock [34]. Traditional approaches have been adopted to construct mutant *E. coli* strain *via* spontaneous adaptation [35] and cloning of exogenous antioxidant genes [36]. Earlier reports suggested that OxyR and RpoS, two major regulators of oxidative stress response in *E. coli*, were either directly or indirectly regulated by CRP [37]. The activation of RpoS (σ^s^) is related with the down regulation of cAMP-CRP complex [37]. The complex was also suggested to stimulate the cleavage of LexA repressor, potentiate ROS generated SOS control and thus transcribe mutagenically important relevant genes upon cell damages [38]. Studies have also revealed that *cya* and *crp* deletion in *E. coli* may increase cellular H_2_O_2_ sensitivity [39]. These findings encouraged us to manipulate relevant *E. coli* response through CRP. Here, we have constructed a CRP library through error-prone PCR [40] and isolated three improved mutants (OM1∼OM3) against oxidative stress *via* enrichment selection (H_2_O_2_). The stress response of the best mutant OM3 and wild type was further analyzed by DNA microarray and validated with quantitative real time reverse transcription PCR (qRT-PCR). Cell lysate of OM3 and WT were tested for antioxidant enzyme activities, namely catalase, alkyl hydroperoxide reductase, and superoxide dismutase.

## Materials and Methods

### Materials

*E. coli* DH5α was procured from Invitrogen (San Diego, USA) and *E. coli Δcrp* strain was obtained according to a previous published protocol [25]. Luria Bertinii (LB) medium (Bacto tryptone (Oxoid) 10 g/l, Yeast extract (Merck) 5 g/l, Sodium Chloride (Merck) 10 g/l) was routinely used for bacterial culture since it has been a popular medium choice for *E. coli* growth under oxidative stress [36], [41]. SOC medium (Yeast extract 5 g/l, Tryptone 20 g/l, NaCl 10 mM, KCl 2.5 mM, MgCl_2_ 10 mM, MgSO_4_ 10 mM, Glucose 20 mM) was used for cultivation of transformed cells. 30% (w/w) hydrogen peroxide (H_2_O_2_) and 2′, 7′-dichlorodihydrofluorescein diacetate (H_2_DCFDA) were purchased from Sigma-Aldrich (St. Louis, MO, US). Restriction enzymes from Fermentas (Burlington, US) and T4 DNA ligase from New England Biolabs (Ipswich, MA, US) were used for cloning and library construction. DNA fragments were purified by QIAquick gel extraction kit (Qiagen, Germany) whenever necessary and plasmid isolation was performed by QIAprep spin miniprep kit from the same manufacturer.

### Cloning and library construction

The native *crp* was amplified *via* error-prone PCR with the following primers: *crp*_sense (5′-gagaggatccataacagaggataaccgcgcatg-3′) and *crp*_anti (5′-agatggtaccaaacaaaatggcgcgctaccaggtaacgcgcca-3′) using Genemorph® random mutagenesis kit from Stratagene (La Zolla, US). The error-prone PCR was performed with 30 ng of pKSCP (containing native *crp* operon) plasmid obtained from our previous studies as template [69], [70], using the following program: 3 min at 95°C, 30 cycles of 45 s at 95°C, 45 s at 62°C followed by 1 min at 72°C, and 10 min at 72°C. The amplified PCR products were purified from 1.2% low-melting agarose gel, double digested with restriction enzymes *Bam* HI and *Kpn* I, and cloned into plasmid pKSCP. The resulting recombinant plasmid was transformed into Δ*crp* competent cells and cultured at 37°C, 200 rpm.

### Mutant selection

The mutant library was cultured in SOC medium at 37°C and 200 rpm for 4 h after electroporation and thence subjected to enrichment selection. In order to select mutants against oxidative stress, H_2_O_2_ was used as stressor and LB medium was fed with increasing concentration of H_2_O_2_. The selection was carried out in 1.5 mM H_2_O_2_ for three repeats and challenged with 2.0 mM H_2_O_2_ during the fourth round. The ‘winners’ were cultured on LB-kanamycin (LB-kan) plates overnight at 37°C. Individual clones were selected randomly from the plates and sequenced to identify amino acid mutations in CRP. The mutated *crp* was re-cloned into fresh pKSCP plasmid and back-transformed to fresh *Δcrp* backgrounds in order to nullify plasmid or genome borne false positives. The pKSCP plasmid containing native *crp* operon was also transformed into *Δcrp* background and is designated as wild type (WT) in this study.

### Mutant growth under stress

The freshly transformed colonies were cultured overnight in LB-kan medium and the overnight inoculums were used to seed cells in fresh LB-kan medium to an OD_600_ value of 0.05. Each clone was cultivated at 37°C, 200 rpm in 0–12 mM H_2_O_2_, 50-ml screw capped centrifuge tube shielded from light. Samples were withdrawn at periodic intervals and cell growth was monitored by measuring the optical density at 600 nm.

Instead of adding H_2_O_2_ at the very beginning, 12 mM H_2_O_2_ was also introduced into the culture after cells reaching mid-log phase (OD_600_ 0.65) and their growth was monitored.

### Tolerance to cumene hydroperoxide

One percent overnight culture was seeded into fresh 10-ml LB-kan medium containing 0.3 mM cumene hydroperoxide. Cell growth was monitored spectrophotometrically at 600 nm.

### Mutant thermotolerance

Stationary phase culture of the mutant and WT was used to inoculate fresh LB-kan media up to an OD_600_ value between 0.05 and 0.06. With the same starting OD_600_, both were allowed to reach stationary phase at 48°C. Cell density was tracked by sampling from the cultures and measuring the OD_600_ values at periodic time intervals.

### Measurement of intracellular reactive oxygen species (ROS) level

The intracellular peroxide level was measured by using ROS sensitive probe 2′, 7′-dichlorodihydrofluorescein diacetate (H_2_DCFDA) as described previously [42]. In brief, both the mutant and WT were grown to OD_600_ 0.6 with or without 4 mM H_2_O_2_. Cells were harvested by centrifugation, washed with 10 mM, pH 7.0 potassium phosphate buffer (PPB), and resuspended in the same buffer. Cells were incubated with 10 µm H_2_DCFDA (dissolved in dimethyl sulfoxide) at 30°C, 200 rpm in darkness for 30 min, harvested, washed again with PPB, and lysed by sonication in darkness. 100-µl cell lysate was pipetted into a 96-well black microplate. Cell fluorescence was measured by a BioTek microplate reader (Winooski, VT, US) with an excitation wavelength at 485 nm and emission at 528 nm. The fluorescence intensity was normalized against total protein concentration measured by Bradford reagent using an Eppendorf biophotometer (Hamburg, Germany).

### DNA microarray

Cells were grown with or without 4 mM H_2_O_2_ to OD_600_ around 0.6∼1 and harvested by centrifugation. RNA was extracted using Qiagen RNeasy kit (Germany) according to manufacturer's instructions. Microarray assay was performed at Genomax Technologies (Singapore). Agilent SurePrint *E. coli* 8×15 K slides were used and Cy3/Cy5 hybridized slides were scanned under Agilent High Resolution Scanner (C-model). Data organization and analysis was performed by Agilent Genespring GX software. Extraction of raw signal data was achieved from TIFF image with Agilent Feature Extraction Software (V10.7.1.1). The expression ratio and *p*-value was calculated based on two biological replicates of each strain under all conditions. A log base 2 transformation was used followed by percentile shift to 75^th^ percentile of each sample (per chip normalization). The normalization was performed by shifting baseline to median of all samples (per gene normalization). *p*-value was calculated using unpaired Student T-Test –with a Benjamini-Horchberg False Discovery Rate (FDR) correction.

The rest of “Materials and Methods” is provided in Materials and Methods S1.

## Results

### Radom mutagenesis library construction and mutant selection

In order to select *E. coli* mutants with elevated tolerance towards oxidative stress, error-prone PCR was performed to introduce mutations to CRP and construct random mutagenesis libraries. Approximately ∼10^5^ clones containing *crp* were obtained after two rounds of error-prone PCR. With the enrichment selection of 1.5 mM∼2.0 mM H_2_O_2_, three mutants (OM1∼OM3) that exhibited better tolerance towards stress were selected from the library. The mutation rate was around 1–3 amino acid substitutions over the CRP open reading frame and the amino acid mutations of OM1∼OM3 are listed in Table 1.

**Table 1:** Amino acid substitutions in OM1∼OM3.

Mutant	Amino acid substitution		
OM1	T127N		
OM2	D138V	T146I	
OM3	F69C	R82C	V139M

### Mutant growth in H_2_O_2_

**Figure 1:**
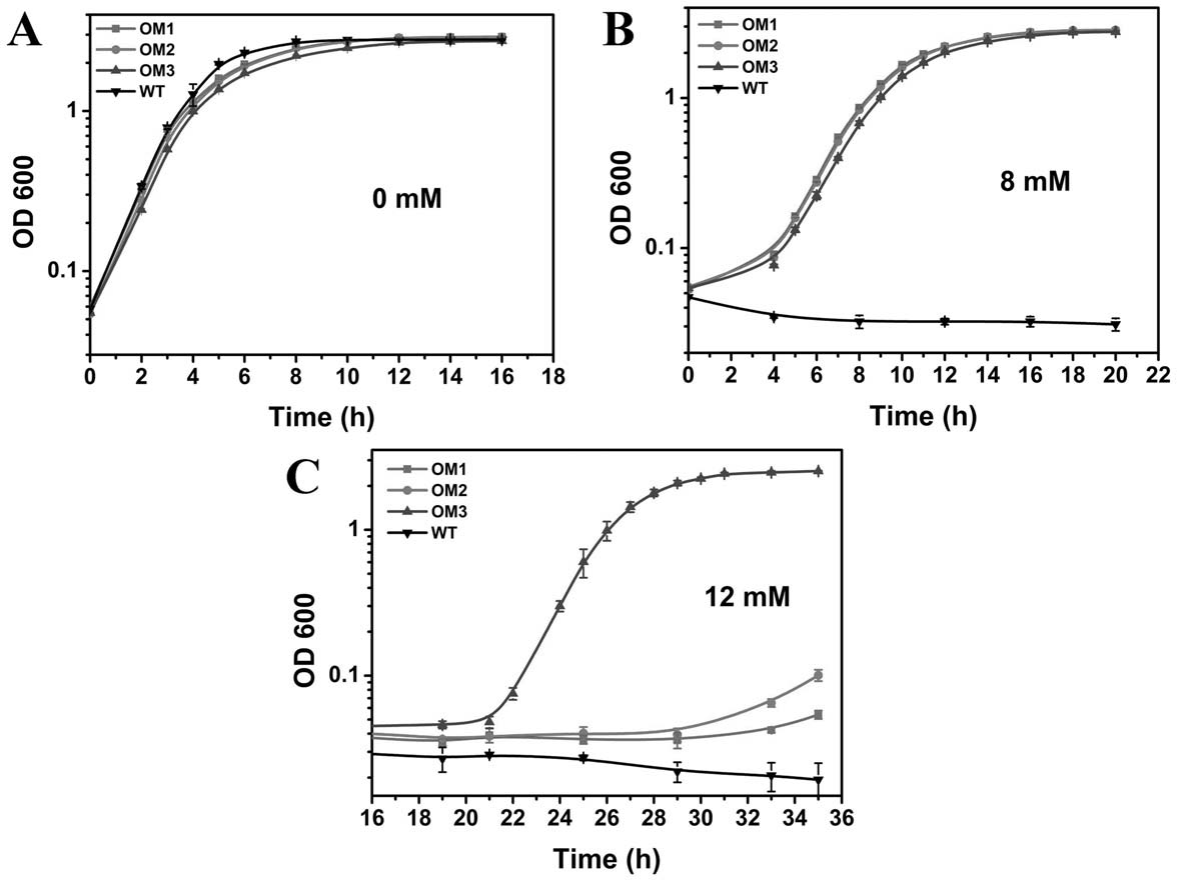
Cell growth in the absence or presence of H_2_O_2_ (A) 0 mM H_2_O_2_, (B) 8 mM H_2_O_2_, (C) 12 mM H_2_O_2_, Cells were cultured in LB-kanamycin medium at 37°C, 200 rpm. Each data point is the mean of three replicates.

Mutant growth was evaluated by subjecting mutants as well as WT in 0 mM to 12 mM H_2_O_2_ (Figure 1). The stability of hydrogen peroxide was confirmed by its absorbance at 240 nm (ε_240_ = 43.6 M^−1^ cm^−1^) during the culturing period (Figure S1). In the absence of H_2_O_2_, all three mutants exhibited similar growth profiles as WT, with the growth rate around 0.31∼0.36 h^−1^ (Figure 1A). With 8 mM H_2_O_2_ present (Figure 1B), all mutants behaved similarly to each other with the growth rate around 0.45 h^−1^, whereas WT exhibited null growth. The cell growth of both *E. coli Δcrp* strain and *Δcrp* strain harboring blank plasmid was also completely inhibited under the same condition (Figure S2). When the pressure was further hiked to 12 mM H_2_O_2_ (Figure 1C), the growth of OM1 and OM2 were hindered completely within the time frame of observation, while OM3 achieved stationary phase OD_600_ of 2.7 with the growth rate of 0.6 h^−1^.

**Figure 2:**
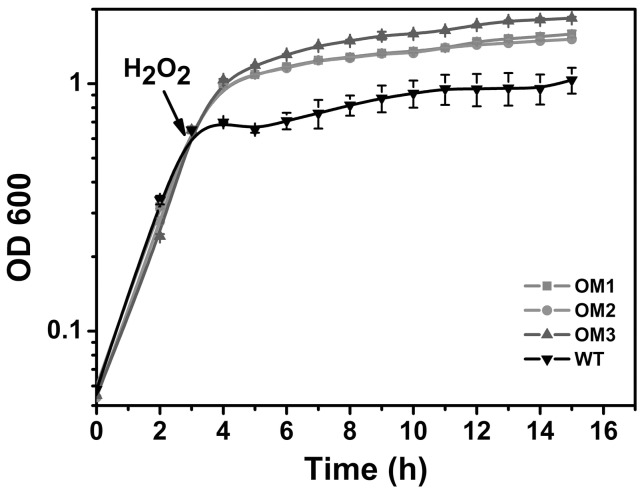
Cell growth profile after the introduction of 12 mM H_2_O_2_ during mid log phase (OD_600_ 0.65). Each data point is the mean of three replicates.

We have also introduced 12 mM H_2_O_2_ into the culture after cells reached mid-log phase in LB-kan medium. OM3 demonstrated the highest stationary phase OD value at 1.84, whereas OM1 and OM2 could only reach ∼1.5 (Figure 2). The inhibition was more prominent in WT as its OD only reached 1.03. Because OM3 displayed the best viability at high H_2_O_2_ concentration, it was chosen for subsequent investigation.

### Mutant thermotolerance and its tolerance to cumene hydroperoxide

**Figure 3:**
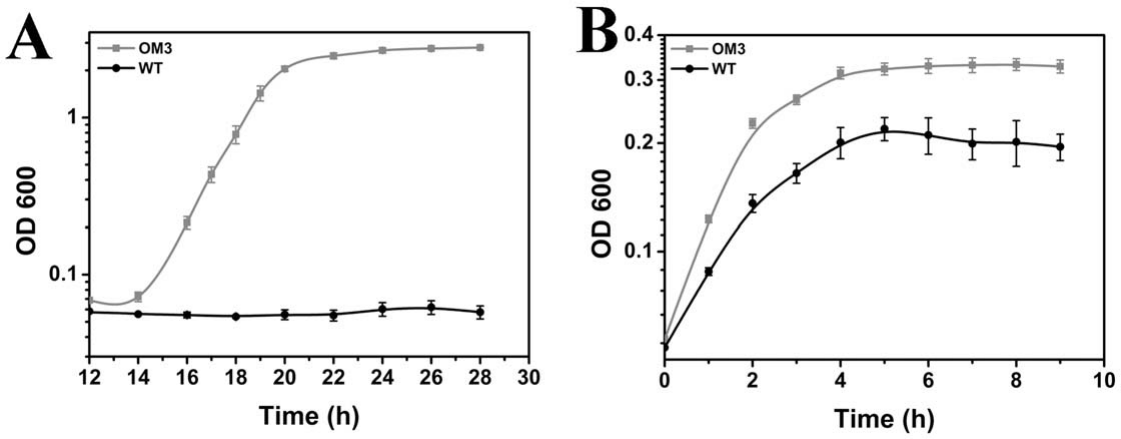
OM3 and WT growth in cumene hydroperoxide or at high temperature (A) 0.3 mM cumene hydroperoxide, (B) 48°C. Cells were grown in LB-kanamycin at 37°C, 200 rpm under above stressors. Each data point is the mean of three replicates.

Since inorganic hydroperoxide H_2_O_2_ was used as oxidative stressor for mutant selection, we further characterized OM3 tolerance against organic hydroperoxide, cumene hydroperoxide. WT growth was completely inhibited in 0.3 mM cumene hydroperoxide while OM3 reached stationary phase at OD_600_ ∼2.8 (Figure 3A). Moreover, earlier publications on the interrelationship between oxidative stress and thermotolerance encouraged us to evaluate the thermotolerance of OM3 [43]. As shown in Figure 3B, OM3 demonstrated better growth (0.52 h^−1^) than WT (0.38 h^−1^) at 48°C.

### DNA microarray analysis and quantitative real time reverse transcription PCR

DNA microarray analysis of OM3 and WT revealed that OM3 had different transcription profile from WT in the presence or absence of oxidative stress, as shown in Table S1 and Table S2 (Gene Expression Omnibus (GEO): GPL13359). In response to oxidative stress, 202 genes in OM3 displayed over twofold up-regulation, while 266 genes showed down-regulation, with the *p*-value threshold less than 0.05. Previous investigation has shown that general stress sigma factor σ^s^ (or RpoS), OxyR and SoxRS regulons play essential roles in regulating *E. coli* oxidative stress response [33], [44]. Here, we found that CRP-regulated genes also went through great expression level changes under oxidative stress—*lamB* (encoding outer membrane protein facilitating diffusion of maltose and other maltodextrins), *malE* (encoding component of maltose ABC transporter), and *cstA* (encoding a carbon starvation protein) were all down-regulated by more than 4.2-fold with H_2_O_2_ treatment (Table 2). Among RpoS-regulated genes, *gadA* (glutamate decarboxylase subunit A) had the maximum fold up-regulation in OM3 (7.76-fold), followed by its family members *gadB* (7.1-fold) and *gadC* (7.02-fold). In addition, increased induction of antioxidant gene *katE* (catalase HP-II, 3.8-fold) was observed. Genes associated with both osmotic as well as oxidative stress tolerance such as *osmC* and *osmY* demonstrated 2.75- and 3.06-fold up-regulation respectively in OM3 compared to WT. OxyR-regulated genes such as *sufABDES* (2.55∼3.48 fold up-regulation) showed enhanced expression level as compared to WT. Without H_2_O_2_ treatment, all of these OxyR-regulated genes revealed less than 2.0-fold change with respect to WT. By contrast, the RpoS-regulated genes exhibited expressional increment, including *gadAB* (4.5-fold), *katE* (2.7-fold), *otsA* (trehalose-6-phosphatase synthase) (2.6-fold) with the threshold *p*<0.05 (Table 3). With or without stress, none of the SoxRS-regulated redox-sensing genes exhibited more than twofold changes as compared to WT. Interestingly, genes regulated by CRP also underwent copious down-regulation (>8.5 fold) in OM3, including *lamB*, *malE*, *malK* (ATP binding component of maltose ABC transporter), which are mainly associated with membrane formation and intracellular transport.

**Table 2:** DNA microarray data of certain genes in OM3 after H_2_O_2_ treatment (*p*<0.05, Log_2_ Fold Change>2.0).

Regulator	b number	Gene	Log_2_ Fold Change
CRP	b4036	*lamB*	−4.780
	b4034	*malE* *	−4.257
	b0598	*cstA* *	−5.093
RpoS	b3517	*gadA* *	7.766
	b1493	*gadB*	7.096
	b1492	*gadC*	7.024
	b1732	*katE* *	3.801
	b1482	*osmC*	2.755
	b4376	*osmY*	3.062
	b1896	*otsA* *	2.996
OxyR	b1684	*sufA*	3.336
	b1683	*sufB*	3.483
	b1681	*sufD*	3.140
	b1680	*sufE*	2.551
	b1679	*sufS*	2.771

* - Analyzed by qRT-PCR (Table S4).

**Table 3:** DNA microarray data of certain endogenous genes in OM3 (*p*<0.05, Log_2_ Fold Change>2.0).

Regulator	b number	Gene	Log_2_Fold Change
CRP	b4036	*lamB*	−8.997
	b4034	*malE* *	−8.930
	b4035	*malK*	−8.502
RpoS	b3517	*gadA* *	4.517
	b1493	*gadB*	4.571
	b1732	*katE* *	2.701
	b1896	*otsA* *	2.581

* - Analyzed by qRT-PCR (Table S4).

qRT-PCR was carried out on ten selected genes to validate the microarray results (Table S3) [33], [45], [46]. Without H_2_O_2_, the expression of *katE*, *gadA*, *crp*, *cya* and *otsA* were all up-regulated in OM3 as compared to WT, whereas *sodA*, *cstA*, *ahpF* and *malE* demonstrated down-regulation, which agreed with the microarray data (Table S4). Under oxidative stress, antioxidant gene expression such as *sodA*, *katE*, *gadA* and *otsA* were elevated in OM3, while *cstA*, *ahpCF*, and *malE* were down-regulated, which also confirmed the microarray results. The only discrepancy we found was that *ahpC* (alkyl hydroperoxide reductase) revealed small activation through microarray under stress but qRT-PCR showed slight down-regulation (Table S4).

### Intracellular reactive oxygen species (ROS) level

**Figure 4:**
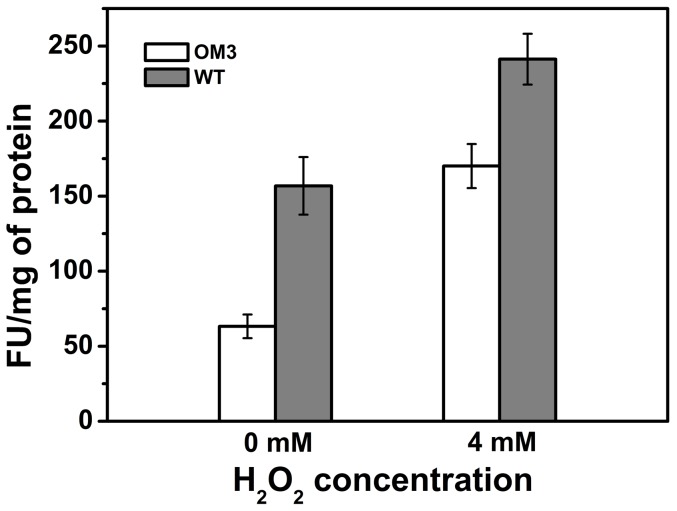
Intracellular ROS level in OM3 and WT with cells treated with or without 4 mM H_2_O_2_. Mid exponential phase grown cells (OD_600_ 0.6) were incubated with 10 µm H_2_DCFDA (dissolved in dimethyl sulfoxide) at 30°C, 200 rpm. The oxidized fluorophore was quantified using excitation wavelength 485 nm and emission wavelength 528 nm. Each data point is the mean of five independent observations.

Oxidative stress in extracellular medium may alter the intracellular peroxide and other ROS level [47] and thus determine cell viability [32]. The normalized fluorescence intensity suggested that the ROS level in OM3 was always lower than that of WT irrespective of growth with or without H_2_O_2_ (Figure 4). In the absence of oxidative stress, OM3 possessed 2.5 times lower intracellular ROS compared to WT. Incubation with 4 mM H_2_O_2_ elevated the free radical level in both strains, diminishing the difference to around 1.4 times.

## Discussion

In this study, we have successfully enhanced *E. coli* oxidative stress tolerance *via* engineering its global regulator CRP. H_2_O_2_ was preferred as the stress-inducing agent in this study and a pool of variants (∼10^5^) was created by error-prone PCR. The library was then screened with H_2_O_2_ and three mutants (OM1∼OM3) with enhanced oxidative stress tolerance were selected. The best mutant OM3 also revealed resistance against cumene hydroperoxide and exhibited thermotolerance.

**Figure 5:**
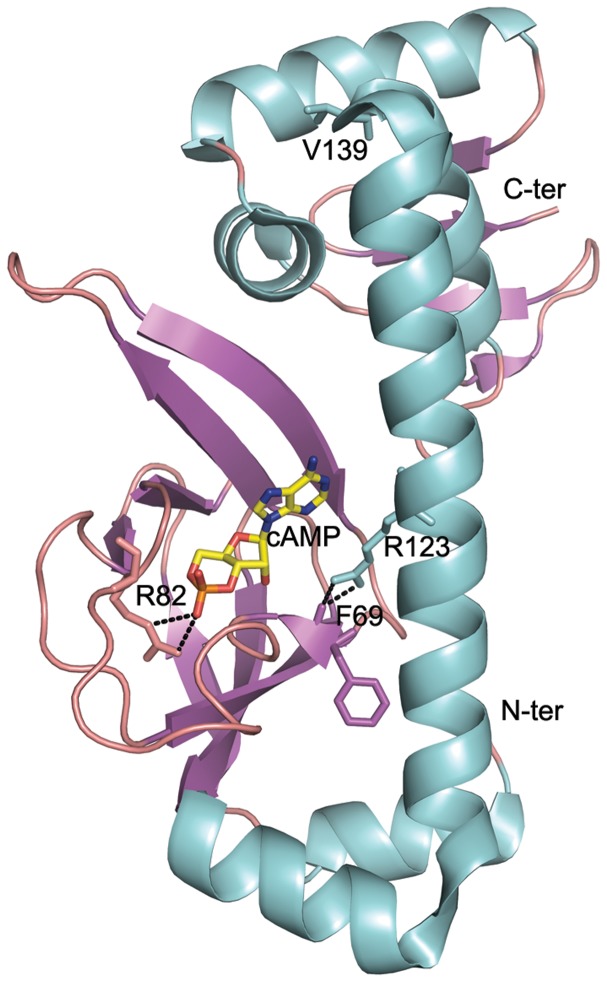
Amino acid mutations in OM3. The main carbonyl of F69 interacts with the amine group of R123. The guanidium group of R82 has the electrostatic interaction with the phosphate group of cAMP. V139 is in the hinge region that participates in the inter-domain interaction between N-terminal cAMP binding domain and the C-terminal DNA binding domain. The structural stereoview was prepared by PyMOL using native CRP structure as template (PDB: 1G6N).

We found that simple modifications to global regulator CRP could result in enhanced strain tolerance towards oxidative stress. As for the best mutant OM3, it obtained three mutations *via* error-prone PCR (F69C, R82C and V139M). F69 is important in conferring CRP conformation, which is reoriented upon cAMP binding with the interaction between F69 and R123 (Figure 5) [48]. R82 sets a pivotal role in cAMP binding due to the electrostatic interaction between the guanidium group of R82 and the electronegative oxygen of the phosphate group of cAMP [49]. V139 is in the hinge region and participates in the interdomain interaction between N- and C-terminals of CRP [50]. The secondary structure of OM3 CRP and native CRP didn't show any significant difference (Figure S3), but the DNA binding properties of the native CRP and OM3 CRP are very different with Class I, Class II and Class III CRP-dependent promoters as tested by reporter gene assay (Figure S4).

Since our target regulator CRP is a global regulator of *E. coli* and can regulate hundreds of genes [51], [52], [53], [54], [55], genome-wide microarray analysis of OM3 and WT in the presence or absence of H_2_O_2_ was performed to reveal the transcription profile change upon modifications to CRP. We found that CRP-regulated genes such as *lamB* and *malEK* showed differential expression in OM3 under either condition. *mal* operon, transcribing genes such as *lamB*, *malE* and *malK* in *E. coli*, is associated with membrane formation and intracellular transport [56]. The repression of these genes in OM3 supported previous reports on the overlap between oxidative stress and acid tolerance response [46]. *mal* operon is regulated by CRP directly [57], implying that genes outside the regulation of the three principle regulators could also play important roles for oxidative stress management in *E. coli*.

Previous publications have suggested that RpoS can regulate the expression of *gadABC*, *katE* and *osmCY* [58], among which *gad* superfamily, namely *gadABC*, displayed maximum up-regulation under stress by microarray (∼7-fold). Glutamate decarboxylase (*gad*) can convert intracellular glutamate to γ-amino butyric acid and is also associated with acid tolerance response of *E. coli* [45]. qRT-PCR result confirmed its upregulation under stress and further enzymatic assay revealed that its activity was 2.9-fold higher in OM3 than in WT under stress (Figure S5A). Strong induction of *katE* (3.8-fold) might lead to a higher amount of catalase in OM3, and thus contributed crucially in the degradation of intracellular H_2_O_2_. qRT-PCR result concurred with microarray data and enzymatic assay proved about 4-fold increased catalase activity in the cell lysate of OM3 as compared to WT (Figure S5B). These findings implied that *katE* could contribute significantly towards OM3 cell protection from oxidative damage. The elevation of *osmCY*, induced upon hyperosmotic stress in OM3 under stress reinforced the paradigm overlap between osmotic stress and oxidative stress [59].

Other major regulons associated with *E. coli* oxidative stress are SoxRS and OxyR, with the latter being suggested as a more specific regulator of H_2_O_2_ responsive pathways [33]. OxyR regulates *suf* operon (*sufABDES*), which is involved in the formation and repair of Fe-S cluster and encodes components of an ATP binding cassette transporter [60]. It was demonstrated by microarray that the expression of *sufABDES* was elevated by more than twofold in OM3 than in WT when treated with H_2_O_2_. Under the same condition, a very minor down-regulation was noted in OxyR-regulated *ahpC* (−0.031-fold) and *ahpF* (−0.483-fold) *via* qRT-PCR. The enzyme assay had also confirmed slightly lower alkyl hydroperoxide reductase activity in the cell lysate of OM3 (Figure S5C). Since AhpC is only active with AhpF present [61], [62], [63], [64], [65], [66], [67], our findings probably have suggested that *ahpCF* are not major players in oxidative stress defense of OM3 [68]. The SoxRS-regulated genes such as *sodA* (manganese-containing superoxide dismutase, SOD) failed to exceed two-fold transcriptional level change under either stressful or normal condition, which was confirmed by qRT-PCR. In addition, little difference was observed in SOD activities between OM3 and WT (Figure S5D), indicating that SOD, similar to alkyl hydroperoxide reductase, did not play an important role in the antioxidant machinery of OM3.

OM3 also exhibited better thermotolerance than WT when exposed to 48°C, which was in cope with the earlier finding that there was an overlap between heat shock and oxidative stress defense mechanism *via* heat shock protease HtrA [69] and heat shock proteins IbpA/B [70]. However, despite the repression of HtrA and IbpA/B or even the chaperones (DnaKJ, GroEL and GroES), the thermotolerance of OM3 was elevated. This phenotypic improvement might be due to the up-regulation of heat shock proteins HtrC and HscA, down-regulation of *sohA* (putative protease of HtrA [71]). The performance of OM3 at 48°C was comparable to *E. coli* MG1655 thermotolerant mutant isolated *via* spontaneous adaptation after two years and 620 generations [72]. In comparison, engineering CRP could greatly shorten the mutant selection period from years to days.

Toxicity of hydrogen peroxide and other oxidative stress is often mediated through generation of intracellular ROS, hence we have investigated relative ROS concentrations in both OM3 and WT. As portrayed in Figure 4, the baseline concentration of endogeneous ROS was 2.5 times lower in OM3 compared to WT in the absence of stress, indicating free radical scavenging system was more active in the mutant than WT. Seaver and Imlay *et al.* reported that Ahp^−^ (Alkyl hydroperoxidase) and Kat^−^ (Catalase) mutants of *E. coli* had a H_2_O_2_ production rate of 14 µM/s [73] whereas that of the wild type was only 1–2 µM/s [74] or 3 µM/s [73]. These findings indicated that the net H_2_O_2_ production of *E. coli* is a direct outcome of intracellular free radical scavenging mechanism. Exertion of stress led to more intracellular ROS accumulation in both OM3 and WT, which was probably due to the increased mass transfer of peroxide into the cells [75]. Since the antioxidant machinery of OM3 might be more active than that of WT, as shown by the elevated expression and activity of catalase, the ROS level in OM3 was lower than that of WT under stress. CRP mediated cellular metabolism could also play an important role in regulating oxidative stress. For instance, ferric uptake regulator protein (Fur) is associated with cell iron metabolism [76], [77]. It helps in protecting intracellular Fe^2+^ ion, which binds with cellular O_2_^−^ radical and depletes intracellular iron pool [78]. Since the cAMP-CRP complex is correlated with Fur activation, it is indirectly related with cellular ROS level [79]. Moreover, thiamine metabolism has been found activated with concomitant up-regulation of CRP-regulated genes in ROS affected cells [80].

Exertion of stress often induces modification to cellular morphology [81]. Interestingly, OM3 underwent no significant change in cell length in the presence or absence of H_2_O_2_ as shown by the micrographs (Figure S6), but the exterior examination of cell revealed that OM3 cell surface has gone rough in either conditions and become even rougher in H_2_O_2_, which might be a morphological response towards oxidative stress.

In this work, OM3 could survive and reach stationary phase of OD_600_∼3.0 in 12 mM H_2_O_2_ whereas the maximal survival limit of WT was 4 mM H_2_O_2_ (data not shown). The only report so far to acquire non-pathogenic *E. coli* tolerance over 12 mM H_2_O_2_ was after adapting cells to glucose starved condition [82]. Metabolic engineering approaches of introducing heterogeneous genes such as *grx* (glutaredoxin) [83], *oscyp2*(rice cyclophilin) [84], and *pprA* (a pleiotropic protein promoting DNA repair in radiation-induced damage) [85] could only help *E. coli* improvise cell tolerance against 5 mM H_2_O_2_, while cloning *Brgr* (glutathione reductase from *Brassica rapa*) [41] helped improve *E. coli* tolerance against 1.5 mM H_2_O_2_. Classical strain engineering approaches using ethyl methane sulphonate (EMS) or UV did not result in significant improvement of *E. coli* tolerance towards oxidative stress [86], [87]. Besides H_2_O_2_, earlier research has improved *E. coli* tolerance towards 0.1–0.4 mM cumene hydroperoxide *via* spontaneous adaptation [88] or chemical mutagen (diethyl sulfate) treatment [89]. Previous studies suggested that a population size ∼10^9^ cells was required to isolate bacterial mutants with tolerance towards cumene hydroperoxide [89].By comparison, we were able to isolate three oxidative stress tolerant mutants from a library size of ∼10^5^. Without pretreatment, the best mutant OM3 exhibited efficient growth against 0.3 mM cumene hydroperoxide, which was comparable to other publications [88]. Hence, together with our previous works, we believe that this isogenic transcriptional engineering approach could provide a promising alternative for *E. coli* strain engineering.

## Supporting Information

Figure S1 H_2_O_2_ absorbance at 240 nm during the culturing period. The experiment was carried out in light shielded environment at 37°C, 200 rpm and H_2_O_2_ concentration was measured spectrophotometrically at 240 nm using molar extinction coefficient of 43.6 M^−1^ cm^−1^.
(TIF)

Figure S2 Cell growth in 6 mM and 8 mM H_2_O_2_. Growth was evaluated in LB-kanamycin (25 µg/ml) medium in light shielded environment. Each data point is the average of two biological replicates.
(TIF)

Figure S3 CD spectra of WT and OM3 CRP. Spectra were obtained in a CHIRASCAN spectropolarimeter with pH 7.2 50 mM PPB buffer as blank. The two spectra were analogous with peaks obtained at 195 and 223 nm. Subsequent deconvolution with K2D2 software revealed that the β-strand percentages in both WT and mutant CRP were close to each other, i.e. 19.53 and 18.65% respectively. Small variation was observed in the relative quantity of α-helix, the percentage being 40.97 in WT and 45.69 in OM3.
(TIF)

Figure S4 DNA binding assay quantified through β-galactosidase activity. The WT and OM3 CRP-pKSCP vectors were co-introduced with distinct pPRO plasmids (pPRO1, pPRO2, and pPRO3) harboring Class I, Class II and Class III CRP-dependent promoters into Δ*crp* strain and the resulting DNA binding was quantified *via* β-galactosidase activity.
(TIF)

Figure S5 Enzyme activity assay. (A) glutamate decarboxylase (GAD) (B) catalase (C) alkyl hydroperoxide reductase (AhpCF) (D) superoxide dismutate (SOD). Each data was the mean of three independent observations.
(TIF)

Figure S6 FESEM micrographs of WT and OM3. (A) WT, 0 mM (B) OM3, 0 mM (C) WT, 4 mM H_2_O_2_ (D) OM3, 4 mM H_2_O_2_.
(TIF)

Materials and Methods S1 Supporting information on materials and methods.
(DOC)

Table S1 Endogenous (untreated) genes in OM3 with expression ratio ≥2 and a *p*-value threshold <0.05.
(DOC)

Table S2 Genes in OM3 with expression ratio ≥2 and a *p*-value threshold <0.05 after H_2_O_2_ treatment.
(DOC)

Table S3 qRT-PCR primers used in this study.
(DOC)

Table S4 DNA microarray and qRT-PCR data comparison of ten selected genes in OM3.
(DOC)

## References

[1] Dalmau M, Lim S, Chen HC, Ruiz C, Wang SW (2008) Thermostability and molecular encapsulation within an engineered caged protein scaffold. Biotechnol Bioeng 101: 654–664. 18814295 10.1002/bit.21988

[2] Dalmau M, Lim S, Wang SW (2009) pH-triggered disassembly in a caged protein complex. Biomacromolecules 10: 3199–3206. 19874026 10.1021/bm900674v

[3] Lim S, Schroder I, Monbouquette HG (2004) A thermostable shikimate 5-dehydrogenase from the archaeon *Archaeoglobus fulgidus* . Fems Microbiol Lett 238: 101–106. 15336409 10.1016/j.femsle.2004.07.023

[4] Lim S, Springstead JR, Yu M, Bartkowski W, Schroder I, et al (2009) Characterization of a key trifunctional enzyme for aromatic amino acid biosynthesis in *Archaeoglobus fulgidus* . Extremophiles 13: 191–198. 19082689 10.1007/s00792-008-0209-z

[5] Schroder I, Vadas A, Johnson E, Lim S, Monbouquette HG (2004) A novel archaeal alanine dehydrogenase homologous to ornithine cyclodeaminase and mu-crystallin. J Bacteriol 186: 7680–7689. 15516582 10.1128/JB.186.22.7680-7689.2004 PMC524889

[6] Huang L, Ching C, Jiang R, Leong S (2008) Production of bioactive human beta-defensin 5 and 6 in *E. coli* by soluble fusion expression. Protein Expr Purif 61: 168–174. 18595735 10.1016/j.pep.2008.05.016

[7] Huang L, Leong S, Jiang R (2009) Soluble fusion expression and characterization of bioactive human beta-defensin 26 & 27. Appl Microbiol Biotechnol 84: 301–308. 19373462 10.1007/s00253-009-1982-z

[8] Zhang F, Rodriguez S, Keasling JD (2011) Metabolic engineering of microbial pathways for advanced biofuels production. Curr Opin Biotechnol 22: 775–783. 21620688 10.1016/j.copbio.2011.04.024

[9] Patnaik R (2008) Engineering complex phenotypes in industrial strains. Biotechnol Prog 24: 38–47. 17914860 10.1021/bp0701214

[10] Conrad TM, Lewis NE, Palsson BO (2011) Microbial laboratory evolution in the era of genome-scale science. Mol Sys Biol 7: 509. 10.1038/msb.2011.42 PMC3159978 21734648

[11] Alper H, Stephanopoulos G (2007) Global transcription machinery engineering: A new approach for improving cellular phenotype. Metab Eng 9: 258–267. 17292651 10.1016/j.ymben.2006.12.002

[12] Zhao X, Jiang R, Bai F (2009) Directed evolution of promoter and cellular transcription machinery and its application in microbial metabolic engineering- a review. Sheng Wu Gong Cheng Xue Bao 25: 1312–1315. 19938472

[13] McArthur GH IV, Fong SS (2010) Toward Engineering Synthetic Microbial Metabolism. J Biomed Biotech 1–10. 10.1155/2010/459760 PMC2796345 20037734

[14] Gottesman S (1984) Bacterial regulation: global regulatory networks. Ann Rev Genetics 18: 415–441. 6099091 10.1146/annurev.ge.18.120184.002215

[15] Lee JY, Sung BH, Yu BJ, Lee JH, Lee SH, et al (2008) Phenotypic engineering by reprogramming gene transcription using novel artificial transcription factors in *Escherichia coli* . Nucleic Acids Res 36: e102. 18641039 10.1093/nar/gkn449 PMC2532725

[16] Park KS, Lee DK, Lee H, Lee Y, Jang YS, et al (2003) Phenotypic alteration of eukaryotic cells using randomized libraries of artificial transcription factors. Nature Biotechnol 21: 1208–1214. 12960965 10.1038/nbt868

[17] Wang J, Zhang Y, Chen Y, Lin M, Lin Z (2012) Global regulator engineering significantly improved *Escherichia coli* tolerances toward inhibitors of lignocellulosic hydrolysates. Biotechnol Bioeng doi: 10.1002/bit.24574. 10.1002/bit.24574 22684885

[18] Chen T, Wang J, Yang R, Li J, Lin M, et al (2011) Laboratory-evolved mutants of an exogenous global regulator, IrrE from *Deinococcus radiodurans*, enhance stress tolerances of *Escherichia coli* . PLoS ONE 6: e16228. 21267412 10.1371/journal.pone.0016228 PMC3022760

[19] Gao C, Wang Z, Liang Q, Qi Q (2010) Global transcription engineering of brewer's yeast enhances the fermentation performance under high-gravity conditions. Appl Microbiol Biotechnol 87: 1821–1827. 20461507 10.1007/s00253-010-2648-6

[20] Klein-Marcuschamer D, Santos CNS, Yu HM, Stephanopoulos G (2009) Mutagenesis of the bacterial RNA polymerase alpha subunit for improvement of complex phenotypes. Appl Environ Microbiol 75: 2705–2711. 19251886 10.1128/AEM.01888-08 PMC2681691

[21] Hong SH, Lee J, Wood TK (2010) Engineering global regulator *Hha* of *Escherichia coli* to control biofilm dispersal. Microb Biotechnol 3: 717–728. 21255366 10.1111/j.1751-7915.2010.00220.x PMC3158428

[22] Hong SH, Wang XX, Wood TK (2010) Controlling biofilm formation, prophage excision and cell death by rewiring global regulator H-NS of *Escherichia coli* . Microb Biotechnol 3: 344–356. 21255333 10.1111/j.1751-7915.2010.00164.x PMC3158429

[23] Perrenoud A, Sauer U (2005) Impact of global transcriptional regulation by ArcA, ArcB, Cra, Crp, Cya, Fnr, and Mlc on Glucose Catabolism in *Escherichia coli* . J Bacteriol 187: 3171–3179. 15838044 10.1128/JB.187.9.3171-3179.2005 PMC1082841

[24] Ma HW, Kumar B, Ditges U, Gunzer F, Buer J, et al (2004) An extended transcriptional regulatory network of *Escherichia coli* and analysis of its hierarchical structure and network motifs. Nucleic Acids Res 32: 6643–6649. 15604458 10.1093/nar/gkh1009 PMC545451

[25] Zhang H, Chong H, Ching CB, Jiang R (2012) Random mutagenesis of global transcription factor cAMP receptor protein for improved osmotolerance. Biotechnol Bioeng 109: 1165–1172. 22179860 10.1002/bit.24411

[26] Zhang H, Chong H, Ching CB, Song H, Jiang R (2012) Engineering global transcription factor cyclic AMP receptor protein of *Escherichia coli* for improved 1-butanol tolerance. Appl Microbiol Biotechnol 94: 1107–1117. 22466954 10.1007/s00253-012-4012-5

[27] Basak S, Hao S, Rongrong J (2012) Error-prone PCR of global transcription factor cyclic AMP receptor protein for enhanced organic solvent (toluene) tolerance. Process Biochem. doi:10.1016/j.procbio.2012.08.006.

[28] O'Donnell A, Bai Y, Bai Z, McNeil B, Harvey LM (2007) Introduction to bioreactors of shake-flask inocula leads to development of oxidative stress in *Aspergillus niger* . Biotechnol Lett 29: 895–900. 17351717 10.1007/s10529-007-9336-3

[29] Zhu C, Ye Q (2003) Selection of acetate-tolerant mutants from *Escherichia coli* DH5alpha and the metabolic properties of mutant DA19. Wei Sheng Wu Xue Bao 43: 460–465. 16276920

[30] Freitas SS, Azzoni AR, Santos JAL, Monteiro GA, Prazeres DMF (2007) On the stability of plasmid DNA vectors during cell culture and purification. Mol Biotechnol 36: 151–158. 17914194 10.1007/s12033-007-0028-y

[31] Storz G, Imlay JA (1999) Oxidative stress. Curr Opin Microbiol 2: 188–194. 10322176 10.1016/s1369-5274(99)80033-2

[32] Greenberg JT, Demple B (1988) Overproduction of peroxide-scavanging enxzymes in *Escherichia coli* suppresses spontaneous mutagenesis and sensitivity to redox-cycling agents in *oxyR* ^−^ mutants. EMBO J 7: 2611–2617. 2847922 10.1002/j.1460-2075.1988.tb03111.x PMC457135

[33] Pomposiello PJ, Demple B (2001) Redox operated genetic switches: the SoxR and OxyR transcription factors. TRENDS Biotechnol 19: 109–114. 11179804 10.1016/s0167-7799(00)01542-0

[34] Nicolaou SA, Gaida SM, Papoutsakis ET (2010) A comparative view of metabolite and substrate stress and tolerance in microbial bioprocessing: From biofuels and chemicals, to biocatalysis and bioremediation. Metab Eng 12: 307–331. 20346409 10.1016/j.ymben.2010.03.004

[35] Demple B, Halbrook J (1983) Inducible repair of oxidative DNA damage in *Escherichia coli* . Nature 304: 466–468. 6348554 10.1038/304466a0

[36] Acuna LG, Calderon IL, Elias AO, Castro ME, Vasquez CC (2009) Expression of the *yggE* gene protects *Escherichia coli* from potassium tellurite-generated oxidative stress. Arch Microbiol 191: 473–476. 19330318 10.1007/s00203-009-0473-z

[37] Barth E, Gora KV, Gebendorfer KM, Settele F, Jakob U, et al (2009) Interplay of cellular cAMP levels, sigma(S) activity and oxidative stress resistance in *Escherichia coli* . Microbiology 155: 1680–1689. 19372151 10.1099/mic.0.026021-0 PMC2848814

[38] Macphee DG (1999) Adaptive mutability in bacteria. J Gen 78: 29–33.

[39] Gonzalez-Flecha B, Demple B (1997) Transcriptional Regulation of the *Escherichia coli oxyR* Gene as a Function of Cell Growth. J Bacteriol 179: 6181–6186. 9324269 10.1128/jb.179.19.6181-6186.1997 PMC179525

[40] Zhang H, Lountos G, Ching C, Jiang R (2010) Engineering of glycerol dehydrogenase for improved activity towards 1,3-butanediol. Appl Microbiol Biotechnol 88: 117–124. 20585771 10.1007/s00253-010-2735-8

[41] Kim IS, Shin SY, Kim YS, Kim HY, Yoon HS (2009) Expression of a Glutathione Reductase from *Brassica rapa* subsp. *pekinensis* Enhanced Cellular Redox Homeostasis by Modulating Antioxidant Proteins in *Escherichia coli* . Mol Cells 28: 479–487. 19936628 10.1007/s10059-009-0168-y

[42] Perez JM, Arenas FA, Pradenas GA, Sandoval JM, Vasquez CC (2008) *Escherichia coli* YqhD Exhibits Aldehyde Reductase Activity and Protects from the Harmful Effect of Lipid Peroxidation-derived Aldehydes. J Biol Chem 283: 7346–7353. 18211903 10.1074/jbc.M708846200

[43] Delaney JM (1990) Requirement of the *Escherichia coli dnaK* gene for thermotolerance and protection against H_2_O_2_ . J Gen Microbiol 136: 2113–2118. 2269877 10.1099/00221287-136-10-2113

[44] Becker-Hapak M, Eisenstark A (1995) Role of *rpoS* in the regulation of glutathione oxidoreductase (gor) in *Escherichia coli* . FEMS Microbiol Lett 134: 39–44. 8593953 10.1111/j.1574-6968.1995.tb07911.x

[45] Castanie-Cornet MP, Penfound TA, Smith D, Elliott JF, Foster JW (1999) Control of acid resistance in *Escherichia coli* . J Bacteriol 181: 3525–3535. 10348866 10.1128/jb.181.11.3525-3535.1999 PMC93821

[46] Maurer LM, Yohannes E, Bondurant SS, Radmacher M, Slonczewski JL (2005) pH regulates genes for flagellar motility, catabolism, and oxidative stress in *Escherichia coli* K-12. J Bacteriol 187: 304–319. 15601715 10.1128/JB.187.1.304-319.2005 PMC538838

[47] Gerard F, Dri A-M, Moreau PL (1999) Role of *Escherichia coli* RpoS, LexA and H-NS global regulators in metabolism and survival under aerobic, phosphate-starvation conditions. Microbiol 145: 1547–1562. 10.1099/13500872-145-7-1547 10439394

[48] Kumar P, Joshi DC, Akif M, Akhter Y, hasnain SE, et al (2010) Mapping conformational transitions in cyclic AMP receptor protein crystal structure and normal-mode analysis of *Mycobacterium tuberculosis* apo-cAMP Receptor Protein. Biophys J 98: 305–314. 20338852 10.1016/j.bpj.2009.10.016 PMC2808490

[49] Moore J, Kantorow M, Vanderzwaag D, McKenney K (1992) *Escherichia coli* cyclic AMP receptor protein mutants provide evidence for ligand contacts important in activation. J Bacteriol 174: 8030–8035. 1334069 10.1128/jb.174.24.8030-8035.1992 PMC207541

[50] Passner JM, Schultz SC, Steitz TA (2000) Modeling the cAMP-induced allosteric transition using the crystal structure of CAP-cAMP at 2.1 Å Resolution. J Mol Biol 304: 847–859. 11124031 10.1006/jmbi.2000.4231

[51] Song H, Payne S, Gray L, You L (2009) Spatiotemporal modulation of biodiversity in a synthetic chemical-mediated ecosystem. Nat Chem Biol 5: 929–935. 19915540 10.1038/nchembio.244 PMC2782429

[52] Song H, Payne S, Tan C, You L (2011) Porgramming microbial population dynamics by engineered cell-cell communication. Biotechnol J 6: 837–849. 21681967 10.1002/biot.201100132 PMC3697107

[53] Song H, Smolen P, Av-Ron E, Baxter DA, Byrne JH (2007) Dynamics of a minimal model of interlocked positive and negative feedback loops of transcriptional regulation by cAMP-response element binding proteins. Biophys J 92: 3407–3424. 17277187 10.1529/biophysj.106.096891 PMC1853161

[54] Song H, You L (2006) Evolving sensitivity. ACS Chem Biol 1: 681. 17184130 10.1021/cb6004596

[55] Yu YY, Chen HL, Yong YC, Kim DH, Song H (2011) Conductive artificial biofilm dramatically enhances bioelectricity production in Shewanella-inoculated microbial fuel cells. Chem Commun (Camb) 47: 12825–12827. 22048750 10.1039/c1cc15874k

[56] Wang YF, Dutzler R, Rizkallah PJ, Rosenbusch JP, Schirmer T (1997) Channel specificity: Structural basis for sugar discrimination and differential flux rates in maltoporin. J Mol Biol 272: 56–63. 9299337 10.1006/jmbi.1997.1224

[57] Chapon C (1982) Role of the catabolite activator protein in the maltose regulon of *Escherichia coli* . J Bacteriol 150: 722–729. 7040340 10.1128/jb.150.2.722-729.1982 PMC216422

[58] Weber H, Polen T, Heuveling J, Wendisch VF, Hengge R (2005) Genome-wide analysis of the general stress response network in *Escherichia coli*:{sigma} S-dependent genes, promoters, and sigma factor selectivity. J Bacteriol 187: 1591–1603. 15716429 10.1128/JB.187.5.1591-1603.2005 PMC1063999

[59] Gunasekera TS, Csonka LN, Paliy O (2008) Genome-wide transcriptional responses of *Escherichia coli* K-12 to continuous osmotic and heat stresses. J Bacteriol 190: 3712–3720. 18359805 10.1128/JB.01990-07 PMC2395010

[60] Zheng M, Wang X, Templeton LJ, Smulski DR, LaRossa RA, et al (2001) DNA microarray-mediated transcriptional profiling of the *Escherichia coli* response to hydrogen peroxide. J Bacteriol 183: 4562–4570. 11443091 10.1128/JB.183.15.4562-4570.2001 PMC95351

[61] Jiang R, Riebel B, Bommarius A (2005) Comparison of Alkyl Hydroperoxide Reductase (AhpR) and water-forming NADH oxidase from *Lactococcus lactis* ATCC 19435. Adv Syn Cat 347: 1139–1146.

[62] Lountos GT, Jiang R, Wellborn WB, Thaler TL, Bommarius AS, et al (2006) The crystal structure of NAD(P)H oxidase from *Lactobacillus sanfranciscensis*: insights into the conversion of O_2_ into two water molecules by the flavoenzyme. Biochemistry 45: 9648–9659. 16893166 10.1021/bi060692p

[63] Wang L, Chong H, Jiang R (2012) Comparison of alkyl hydroperoxide reductase and two water-forming NADH oxidases from *Bacillus cereus* ATCC 14579. Appl Microbiol Biotechnol doi: 10.1007/s00253-012-3919-1. 10.1007/s00253-012-3919-1 22311647

[64] Wang L, Wei L, Chen Y, Jiang R (2010) Specific and reversible immobilization of NADH oxidase on functionalized single-walled carbon nanotubes. J Biotechnol 150: 57–63. 20630484 10.1016/j.jbiotec.2010.07.005

[65] Wang L, Xu R, Chen Y, Jiang R (2011) Activity and stability comparison of immobilized NADH oxidase on multi-walled carbon nanotubes, carbon nanospheres, and single-walled carbon nanotubes. J Mol Catal B: Enz 69: 120–126.

[66] Wang L, Chen Y, Jiang R (2012) Nanoparticle-supported consecutive reactions catalyzed by alkyl hydroperoxide reductase. J Mol Catal B: Enz 76: 9–14.

[67] Wang L, Zhang H, Ching C, Chen Y, Jiang R (2012) Nanotube-supported bioproduction of 4-hydroxy-2-butanone via in situ cofactor regeneration. Appl Microbiol Biotechnol 94: 1233–1241. 22116631 10.1007/s00253-011-3699-z

[68] Jiang R, Bommarius A (2004) Hydrogen peroxide-producing NADH oxidase (nox-1) from *Lactococcus lactis* . Tetrahedron: Asymmetry 15: 2939–2944.

[69] Skorko-Glonek J, Zurawa D, Kuczwara E, Wozniak M, Wypych Z, et al (1999) The *Escherichia coli* heat shock protease HtrA participates in defense against oxidative stress. Mol Gen Genet 262: 342–350. 10517331 10.1007/s004380051092

[70] Matuszewska E, Kwiatkowska J, Kuczyńska-Wiśnik D, Laskowska E (2008) *Escherichia coli* heat-shock proteins IbpA/B are involved in resistance to oxidative stress induced by copper. Microbiology 154: 1739–1747. 18524928 10.1099/mic.0.2007/014696-0

[71] Baird L, Georgopoulos C (1990) Identification, Cloning, and Characterization of the *Escherichia coli sohA* Gene, a Suppressor of the *htrA* (*degP*) Null Phenotype. J Bacteriol 172: 1587–1594. 2407727 10.1128/jb.172.3.1587-1594.1990 PMC208636

[72] Rudolph B, Gebendorfer KM, Buchner J, Winter J (2010) Evolution of *Escherichia coli* for Growth at High Temperatures. J Biol Chem 285: 19029–19034. 20406805 10.1074/jbc.M110.103374 PMC2885180

[73] Seaver LC, Imlay JA (2001) Alkyl hydroperoxide reductase is the primary scavanger of endogeneous hydrogen peroxide in *Escherichia coli* . J Bacteriol 183: 7173–7181. 11717276 10.1128/JB.183.24.7173-7181.2001 PMC95566

[74] Gonzalez-Flecha B, Demple B (1997) Homeostatic regulation of intracellular hydrogen peroxide concentration in aerobically growing *Escherichia coli* . J Bacteriol 179: 382–388. 8990289 10.1128/jb.179.2.382-388.1997 PMC178707

[75] Seaver LC, Imlay JA (2001) Hydrogen peroxide fuxes and compartmentalization inside growing *Escherichia coli* . J Bacteriol 183: 7182–7189. 11717277 10.1128/JB.183.24.7182-7189.2001 PMC95567

[76] Hantke K (2001) Iron and metal regulator in bacteria. Curr Opin Microbiol 4: 172–177. 11282473 10.1016/s1369-5274(00)00184-3

[77] Vassinova N, Kozyrev D (2000) A method for direct cloning of *fur*-regulated genes: identification of seven new *fur*-regulated loci in *Escherichia coli* . Microbiology 146: 3171–3182. 11101675 10.1099/00221287-146-12-3171

[78] Touati D, Jacques M, Tardat B, Bouchard L, Despied S (1995) Lethal oxidative damage and mutagenesis are generated by iron in Δ*fur* mutants of *Escherichia coli* : protective role of superoxide dismutase. J Bacteriol 177: 2305–2314. 7730258 10.1128/jb.177.9.2305-2314.1995 PMC176885

[79] Campoy S, Jara M, Busquets N, Prez de Rozas AM, Badiola I, et al (2002) Intracellular cyclic AMP concentration is decreased in *Salmonella typhimurium fur* mutants. Microbiology 148: 1039–1048. 11932449 10.1099/00221287-148-4-1039

[80] Fukui K, Wakamatsu T, Agari Y, Masui R, Kuramitsu S (2011) Inactivation of the DNA repair genes *mutS*, *mutL* or the anti-recombination gene *mutS2* leads to activation of vitamin B1 biosynthesis genes. PLos ONE 6: e19053. 21552516 10.1371/journal.pone.0019053 PMC3084264

[81] Patil S, Valdramidis VP, Karatzas KAG, Cullen PJ, Bourke P (2011) Assessing the microbial oxidative stress mechanism of ozone treatment through the responses of *Escherichia coli* mutants. J Appl Microbiol 111: 136–144. 21457413 10.1111/j.1365-2672.2011.05021.x

[82] Jenkins DE, Schultz JE, Matin A (1988) Starvation-induced cross protection against heat or H_2_O_2_ challenge in *Escherichia coli* . J Bacteriol 170: 3910–3914. 3045081 10.1128/jb.170.9.3910-3914.1988 PMC211389

[83] Li M, Huang W, Yang Q, Liu X, Wu Q (2005) Expression and oxidative stress tolerance studies of glutaredoxin from cyanobacterium *Synechocystis* sp. PCC 6803 in *Escherichia coli* . Protein Expr Purif 42: 85–91. 15882949 10.1016/j.pep.2005.03.027

[84] Kumari S, Singh P, Singla-Pareek SL, Pareek A (2009) Heterologous expression of a salinity and developmentally regulated rice cyclophilin gene (*OsCyp2*) in *E. coli* and *S. cerevisiae* confers tolerance towards multiple abiotic stresses. Mol Biotechnol 42: 195–204. 19214808 10.1007/s12033-009-9153-0

[85] Kota S, Misra HS (2006) PprA: A protein implicated in radioresistance of *Deinococcus radiodurans* stimulates catalase activity in *Escherichia coli* . Appl Microbiol Biotechnol 72: 790–796. 16586106 10.1007/s00253-006-0340-7

[86] Mulder MA, Nair S, Abratt VR, Zappe H, Steyn LM (1999) Involvement of the N- and C-terminal domains of *Mycobacterium tuberculosis* KatG in the protection of mutant *Escherichia coli* against DNA-damaging agents. Microbiology 145: 2011–2021. 10463167 10.1099/13500872-145-8-2011

[87] Prieto-Alamo M-J, Abril M, Pueyo C (1993) Mutagenesis in *Escherichia coli* K-12 mutants defective in superoxide dismutase or catalase. Carcinogenesis 14: 237–244. 8382113 10.1093/carcin/14.2.237

[88] Asad NR, Asad L, Silva AB, Felzenszwalb I, Leitao C (1998) Hydrogen peroxide induces protection against lethal effects of cumene hydroperoxide in *Escherichia coli* cells: an Ahp dependent and OxyR independent system? Mut Res-DNA Repair 407: 253–259. 9653451 10.1016/s0921-8777(98)00010-x

[89] Storz G, Jacobson FS, Tartaglia LA, Morgan RW, Silveira LA, et al (1989) An Alkyl Hydroperoxide Reductase Induced by Oxidative Stress in *Salmonella typhimurium* and *Escherichia coli* : Genetic Characterization and Cloning of *ahp* . J Bacteriol 171: 2049–2055. 2649484 10.1128/jb.171.4.2049-2055.1989 PMC209856

