# Supplementary figures and images for: Enhancing E. coli Tolerance towards Oxidative Stress via Engineering Its Global Regulator cAMP Receptor Protein (CRP)
Source: PLoS One. 2012 Dec 14;7(12):e51179. doi: 10.1371/journal.pone.0051179 (PMC3522674; doi:10.1371/journal.pone.0051179)

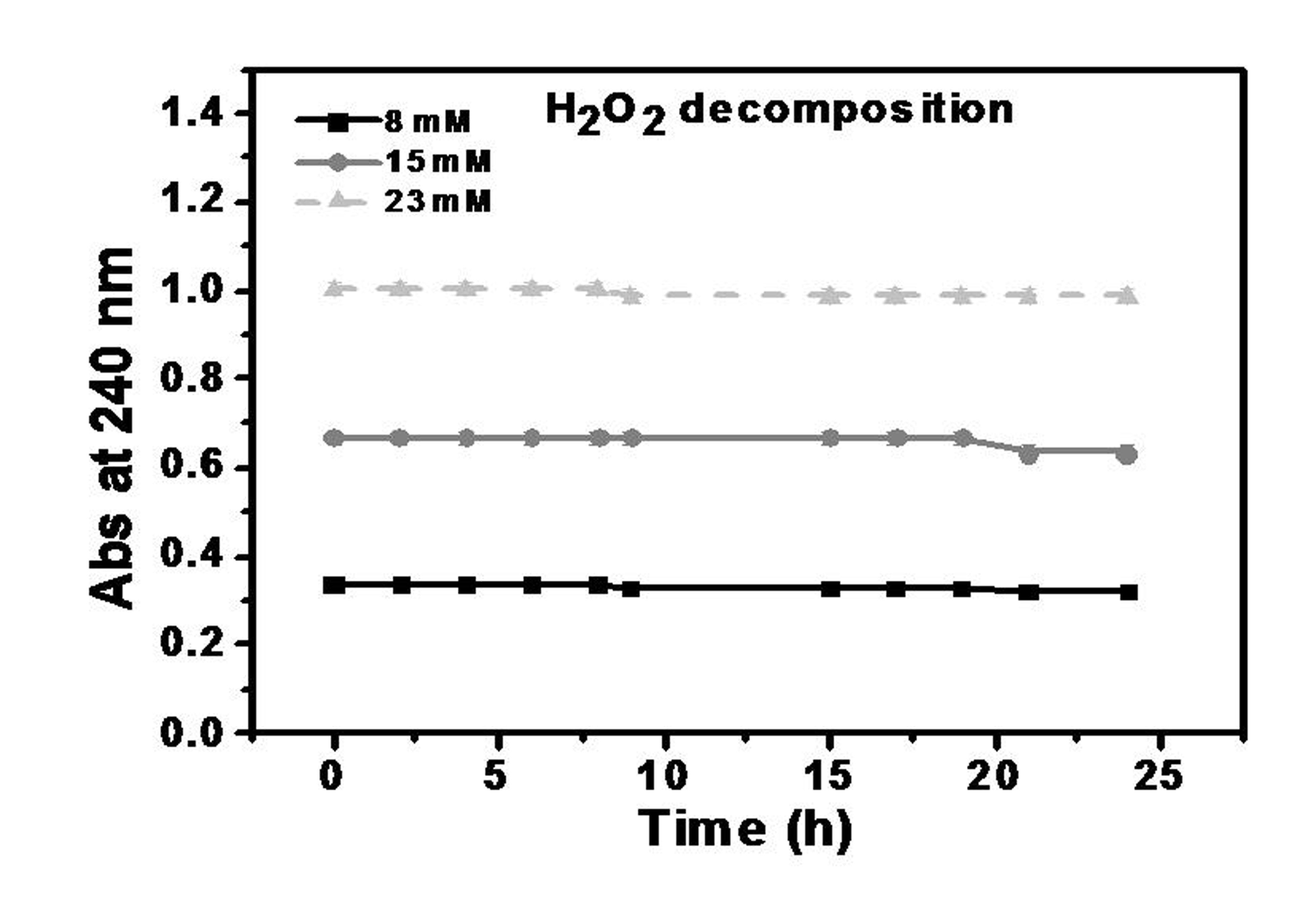

Supplement: Figure S1 — H2O2 absorbance at 240 nm during the culturing period. The experiment was carried out in light shielded environment at 37°C, 200 rpm and H2O2 concentration was measured spectrophotometrically at 240 nm using molar extinction coefficient of 43.6 M−1 cm−1. (TIF) [file pone.0051179.s001.tif]

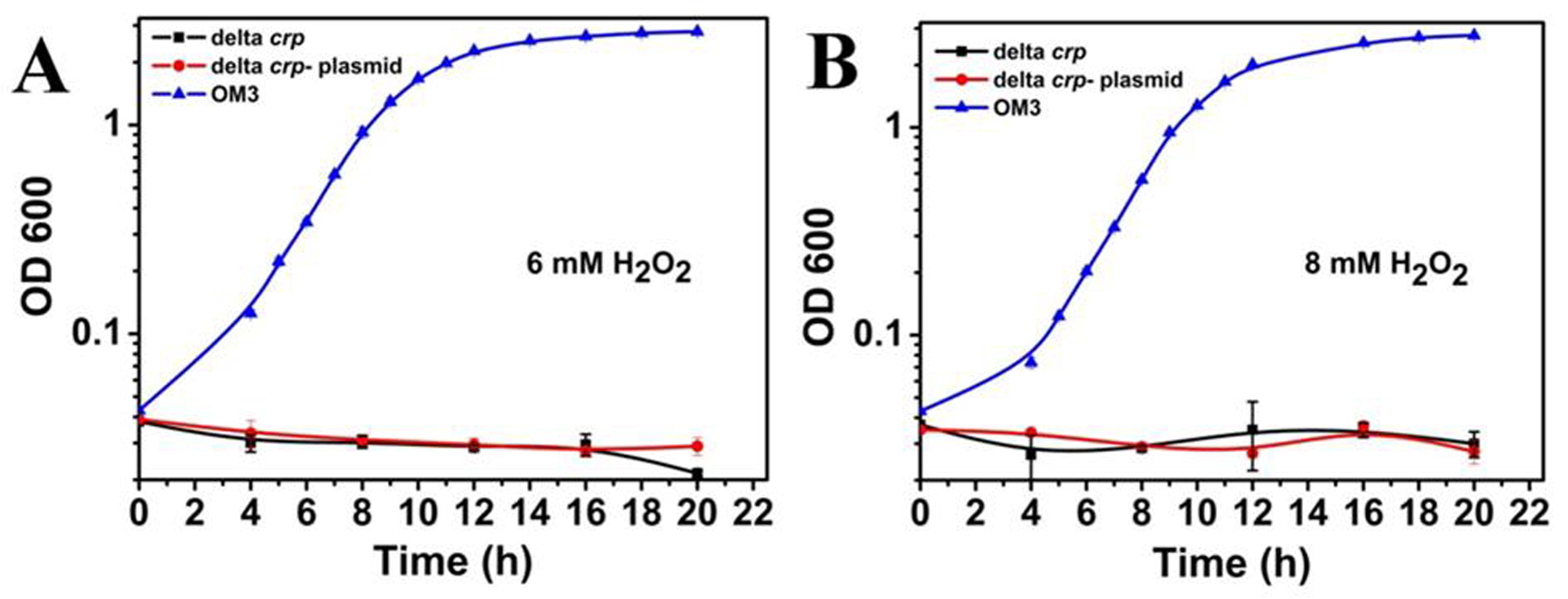

Supplement: Figure S2 — Cell growth in 6 mM and 8 mM H2O2. Growth was evaluated in LB-kanamycin (25 µg/ml) medium in light shielded environment. Each data point is the average of two biological replicates. (TIF) [file pone.0051179.s002.tif]

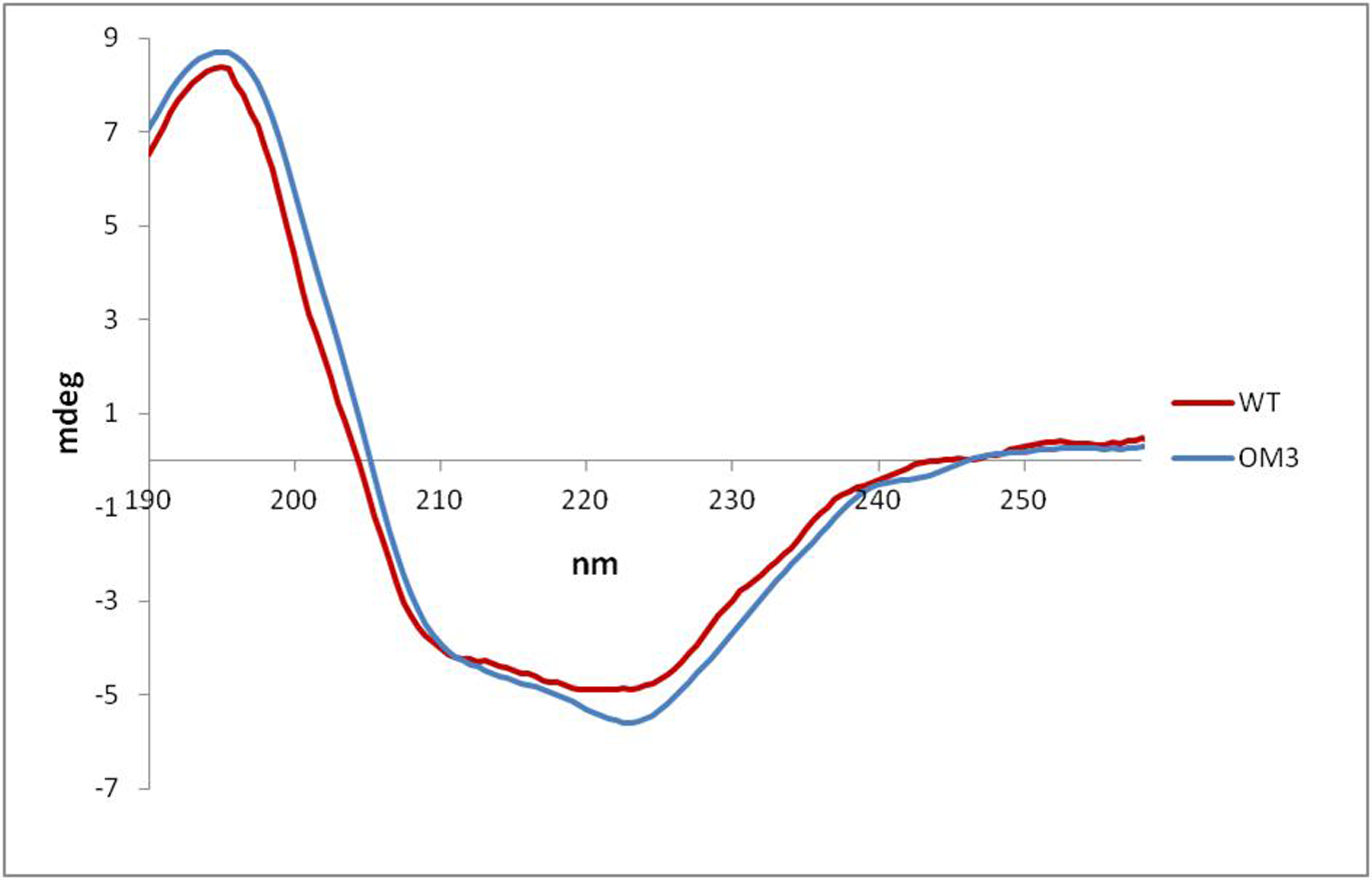

Supplement: Figure S3 — CD spectra of WT and OM3 CRP. Spectra were obtained in a CHIRASCAN spectropolarimeter with pH 7.2 50 mM PPB buffer as blank. The two spectra were analogous with peaks obtained at 195 and 223 nm. Subsequent deconvolution with K2D2 software revealed that the β-strand percentages in both WT and mutant CRP were close to each other, i.e. 19.53 and 18.65% respectively. Small variation was observed in the relative quantity of α-helix, the percentage being 40.97 in WT and 45.69 in OM3. (TIF) [file pone.0051179.s003.tif]

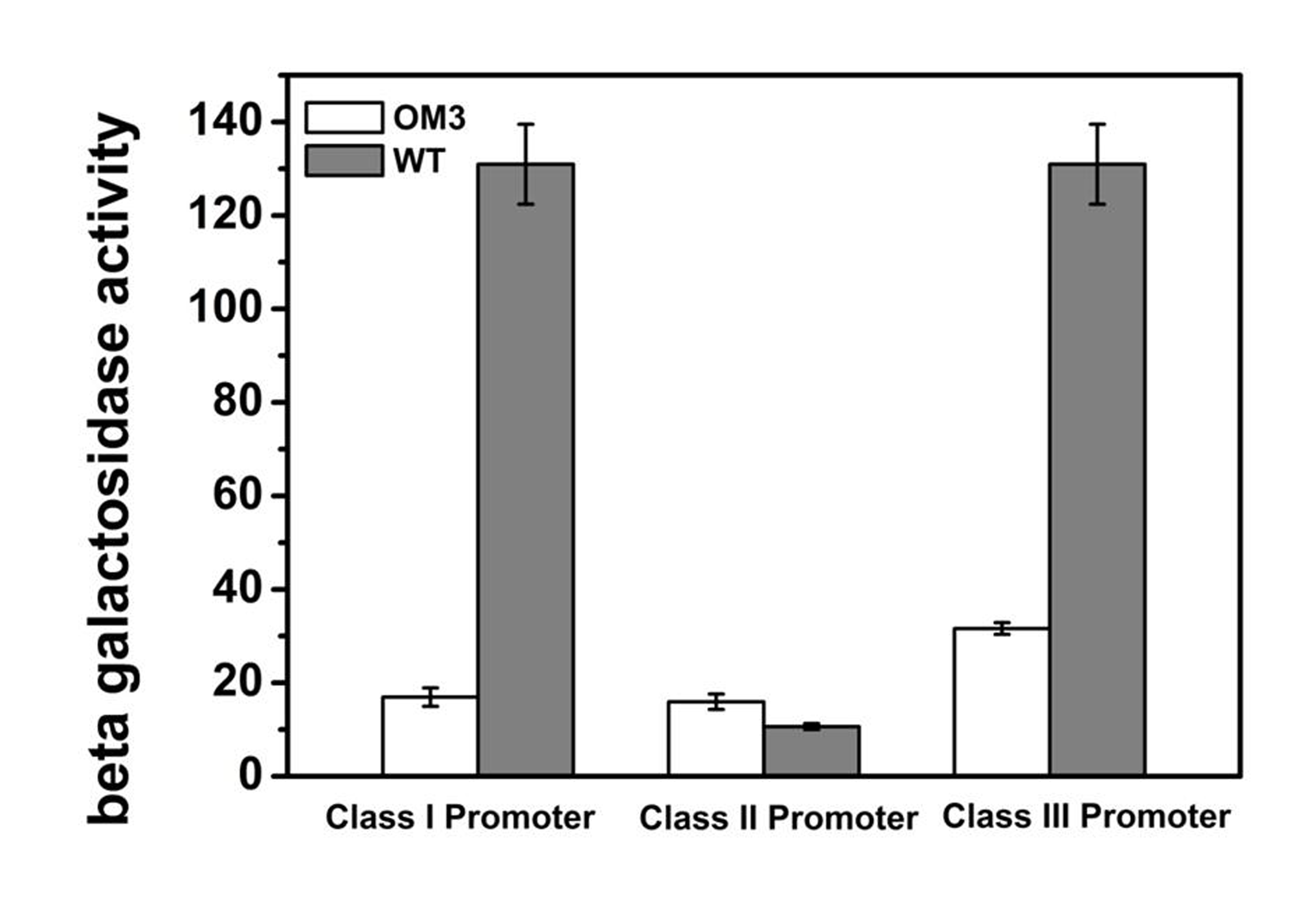

Supplement: Figure S4 — DNA binding assay quantified through β-galactosidase activity. The WT and OM3 CRP-pKSCP vectors were co-introduced with distinct pPRO plasmids (pPRO1, pPRO2, and pPRO3) harboring Class I, Class II and Class III CRP-dependent promoters into Δcrp strain and the resulting DNA binding was quantified via β-galactosidase activity. (TIF) [file pone.0051179.s004.tif]

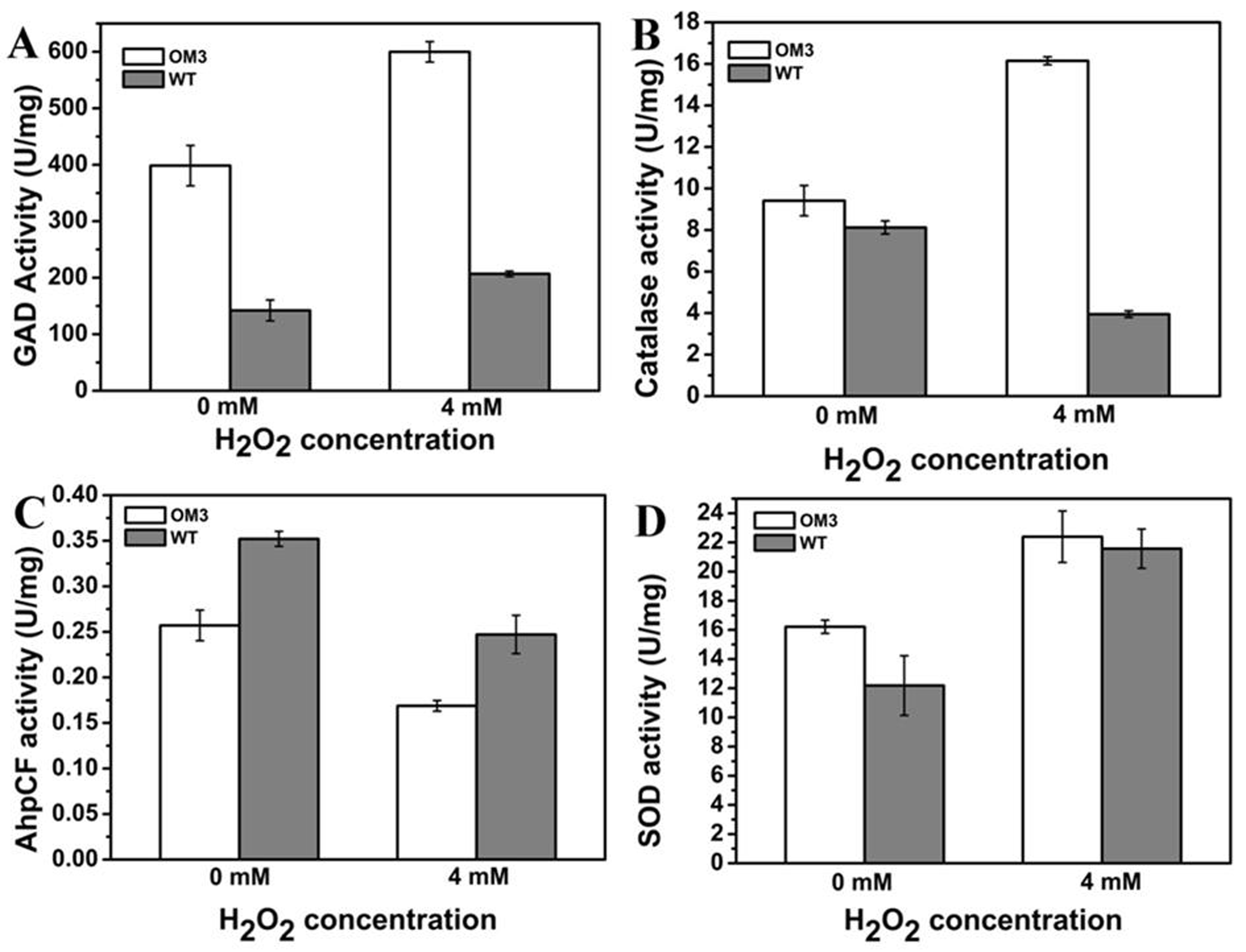

Supplement: Figure S5 — Enzyme activity assay. (A) glutamate decarboxylase (GAD) (B) catalase (C) alkyl hydroperoxide reductase (AhpCF) (D) superoxide dismutate (SOD). Each data was the mean of three independent observations. (TIF) [file pone.0051179.s005.tif]

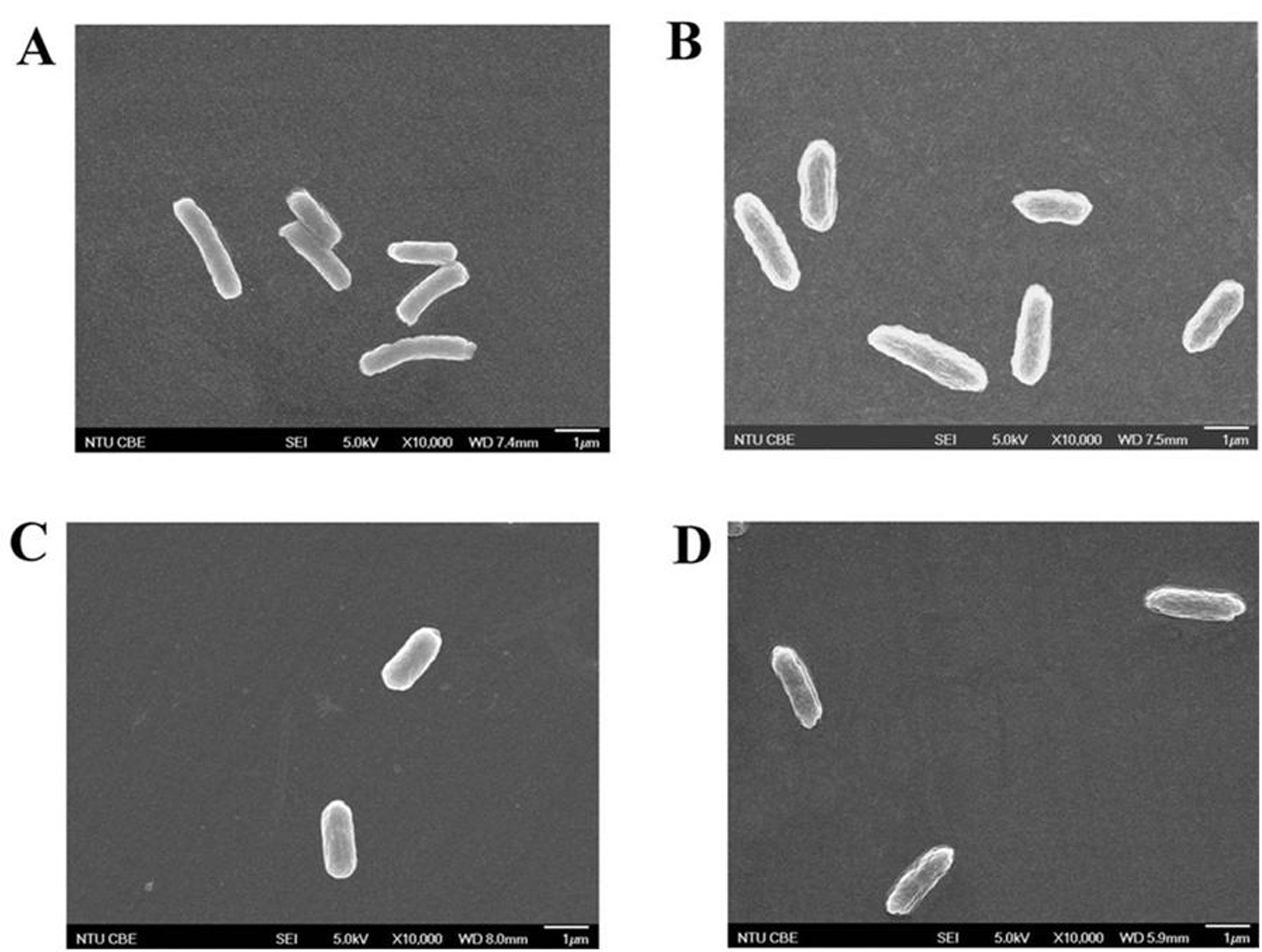

Supplement: Figure S6 — FESEM micrographs of WT and OM3. (A) WT, 0 mM (B) OM3, 0 mM (C) WT, 4 mM H2O2 (D) OM3, 4 mM H2O2. (TIF) [file pone.0051179.s006.tif]
