# Supplementary material for: Enhancing E. coli Tolerance towards Oxidative Stress via Engineering Its Global Regulator cAMP Receptor Protein (CRP)
Source: PLoS One. 2012 Dec 14;7(12):e51179. doi: 10.1371/journal.pone.0051179 (PMC3522674; doi:10.1371/journal.pone.0051179)
Supplement: Materials and Methods S1 — Supporting information on materials and methods. (DOC) [file pone.0051179.s007.doc]

**Supplementary Materials and Methods**

**Field Emission Scanning Electron Microscopy (FESEM)**

Cells were first grown in the absence or presence of 4 mM H_2_O_2_ and cell pellets were harvested by centrifugation, washed with ice-cold 0.9% NaCl, and thereafter fixed with 2.0% glutaraldehyde overnight. The fixed cells were harvested and washed with DI water to remove all residual glutaraldehyde and NaCl. Cells were then resuspended in DI water, and 2-3 µl cell suspension was used to coat a glass slide. The cells were dehydrated with increasing concentrations (10%-100%) of ethanol and dried overnight at 37°C. Cell surface was coated with platinum sputter for 75 s and observed with a JEOL JSM 6701 Microscope (JEOL, Japan).

**Quantitative real time reverse transcription PCR (qRT-PCR)**

cDNA was synthesized from cellular RNA extracted from both OM3 and WT grown in the presence or absence of H_2_O_2_. It was performed with iScript cDNA synthesis kit (Bio-Rad, CA, US) using 500 ng cellular RNA as template. Ten genes were chosen for qRT-PCR analysis to validate the microarray results. The primers used are listed in SI Table S3. qRT-PCR was carried out using Bio-Rad iQ5 thermocycler with the following programme: 3 min at 95 ^o^C, 40 cycles at 95 ^o^C for 15 s, followed by 60 ^o^C for 30 s. 2^-ΔΔCt^ method was used for gene expression analysis. The observations were based on two biological replicates.

**Enzyme activity assay**

For all enzyme activity assay, fresh cells were cultivated up to OD_600_~0.6, harvested by centrifugation at 1900 × g for 15 min, lysed by ultrasonication and cell lysate was subjected to assay under specific conditions. Cellular protein concentration was determined by Bradford assay.

**i) Superoxide Dismutase (SOD)**

SOD activity assay was performed by pyrogallol autoxidation method as described before [1]. Briefly, pH 8.2, 50 mM Tris-Cl buffer with 1 mM EDTA was used as reaction medium. Cell lysate containing 40~60 µg cellular proteins was added to 0.2 mM pyrogallol (dissolved in pH 6.5, 50 mM PPB) to initiate the reaction, and the absorbance decrease of pyrogallol was monitored at 420 nm. The percentage inhibition of pyrogallol autoxidation was calculated by the following formula:

% inhibition of pyrogallol autoxidation= [1-(ΔA/ΔA_max_)] x 100, where

ΔA= Absorbance change due to pyrogallol autoxidation in the sample reaction system

ΔA_max_ = Absorbance change due to pyrogallol autoxidation in the control (without cell lysate)

One unit of SOD activity was defined as the amount required for inhibiting pyrogallol autoxidation by 50% per min.

**ii) Catalase assay**

Catalase assay was carried out according to a previous protocol with modifications [2]. About 40~60 µg cellular protein and 10 mM H_2_O_2_ was added to pH 7.2, 50 mM PPB reaction medium. The absorbance drop of H_2_O_2_ at 240 nm (ɛ_240_ = 43.6 mM^-1^ cm^-1^ ) was recorded at 25ºC [3]. One unit of catalase activity was defined as the µmoles of H_2_O_2_ decomposed per min.

**iii) Alkyl hydroperoxide reductase (AhpCF) assay**

As per the method published earlier [4], the reaction system comprised of 1mM cumene hydroperoxide, 240 µM NADH, 40~60 µg cellular protein dissolved in pH 7.2, 50 mM PPB containing 0.5 mM EDTA. The reaction was monitored at 340 nm (NADH, ɛ: 6.22 x 10^3^ M^-1^ cm^-1^) [5]. One unit of alkyl hydroperoxide reductase activity was defined as the µmoles of NADH consumed per min.

**iv) Glutamate decarboxylase (GAD) assay**

The assay was followed as described before [6]. Briefly, the analysis was based on the pH indicator assay where the color of Bromocresol green can change within pH 3.8-5.4. The reaction system was comprised of 10 mM monosodium salt of L-glutamic acid (MSG), 10 mM pyridoxal 5/- phosphate, 70 µm Bromocresol green as pH indicator, 60 µg of cell protein and 20 mM acetate buffer (pH 4.8) as medium,. The reaction was carried out at 40°C and the absorbance change was monitored at 620 nm. One unit of glutamate decarboxylase activity was defined as µmoles of MSG consumed per min.

**DNA binding properties of the native CRP and OM3 CRP**

DNA binding was performed *via* reporter gene assay using a yeast β-galactosidase assay kit (Thermo Scientific, USA) according to manufacturer’s instructions. The expression of the reporter gene is dependent upon the specific interaction between the DNA-binding domain of promoters and CRP. Briefly, the CRP-dependent promoters (Class I, Class II, and Class III) had been cloned into pPR9TT plasmid [7] to construct recombinant plasmids pPRO1, pPRO2, and pPRO3 respectively. For the Class III promoter, *acs* (acetate CoA ligase (AMP forming)) promoter was used [8]. Each pPRO plasmid was co-transformed into ∆*crp* strain with plasmid pKSCP harbouring either native or OM3 *crp*. The colonies were picked and cultured to obtain mid log phase cells (OD_600_ = 0.6 ~ 0.7). 350 µl cell culture was added with equal volume of working solution, incubated at 37°C for galactosidase expression and subsequent reaction. The reaction was finally stopped by the addition of stop solution and the resulting absorbance of yellow colored *o*-nitrophenol obtained from *o*-nitrophenyl-β-D galactopyranoside (ONPG) was measured at 420 nm. A control without cells was used as blank. The activity of β-galactosidase was calculated as

**=** β-galactosidase activity

1000 × A_420_

t × V ×OD_600_

where A_420_ = absorbance at 420 nm

t = time of reaction in mins

v= volume of cells in ml used in reaction

**Secondary structure**

The *crp* of WT and OM3 was amplified by PCR with the following primers: *crp*_sense- 5/- ATA GGA TCC TAT GCA TCA TCA CCA TCA CCA CAT GGT GCT TGG CAA ACC - 3/ ; *crp*_ antisense: 5/- AAC GCT CGA GTT AAC GAG TGC CGT AAA CGA CGA TGG TTT TAC CGT G – 3/. The PCR products were double digested with *Bam* HI and *Xho* I and subcloned into pET 21b (+) vector (Novagen, USA). The open reading frame was verified by DNA sequencing and the recombinant plasmid was subsequently transformed into *E. coli* BL21 (DE3) for protein over-expression with 0.3 mM IPTG. Both native CRP and OM3 CRP were purified with His-Gravi Trap column (GE Healthcare, UK) and stored in 50 mM, pH 7.2 potassium phosphate buffer (PPB).

The secondary structure of protein was acquiesced by steady state circular dichorism (CD), the measurement being carried out at far UV range (180-260 nm) using a CHIRASCAN spectropolarimeter (Applied Photophysics, UK). Spectroscopic analysis was performed in 60 µl quartz cell (Hellma) at 22°C with a step resolution of 1 nm. The final protein spectra were obtained by auto-subtraction of the baseline, analyzed by Chirascan 4.2.12 software and was used as input for *in-silico* secondary structure prediction (Protein Eng 1992; 5: 191-195). The data points were finally deconvoluted using K2D2 software, freely available online.

**References**

1. Marklund S, Marklund G (1974) Involvement of the Superoxide Anion Radical in the Autoxidation of Pyrogallol and a Convenient Assay for Superoxide Dismutase. Eur J Biochem 47: 469-474.

2. Acuna LG, Calderon IL, Elias AO, Castro ME, Vasquez CC (2009) Expression of the *yggE* gene protects *Escherichia coli* from potassium tellurite-generated oxidative stress. Arch Microbiol 191: 473-476.

3. Beers RFJ, Sizer I (1951) A spetrophotometric method for measuring the breakdown of hydrogen peroxide by catalase. J Biol Chem 195: 133-140.

4. Hillas PJ, del Alba FS, Oyarzabal J, Wilks A, de Montellano PRO (2000) The AhpC and AhpD Antioxidant Defense System of *Mycobacterium tuberculosis*. J Biol Chem 275: 18801-18809.

5. Baez M, Rodriguez PH, Babul J, Guixe V (2003) Structural and functional roles of Cys-238 and Cys-295 in *Escherichia coli* phosphofructokinase-2. Biochem J 376: 277-283.

6. Yu K, Hu S, Huang J, Mei LH (2011) A high throughput colorimetric assay to measure the activity of glutamate decarboxylase Enzyme Microb technol 49: 272-276.

7. Santos PM, Di BI, Blatny JM, Zennaro E, Valla S (2001) New broad-host-range promoter probe vectors based on the plasmid RK2 replicon. FEMS Microbiol Lett 195: 91-96

8. Sclavi B, Beatty CM, Thach DS, Fredericks CE, Buckle M, et al. (2007) The multiple roles of CRP at the complex *acs* promoter depend on activation region 2 and IHF. Mol Microbiol 65: 425-440
