# Supplementary material for: Enhancing E. coli Tolerance towards Oxidative Stress via Engineering Its Global Regulator cAMP Receptor Protein (CRP)
Source: PLoS One. 2012 Dec 14;7(12):e51179. doi: 10.1371/journal.pone.0051179 (PMC3522674; doi:10.1371/journal.pone.0051179)
Supplement: Table S1 — Endogenous (untreated) genes in OM3 with expression ratio ≥2 and a p-value threshold <0.05. (DOC) [file pone.0051179.s008.doc]

**TABLE S1** Endogenous (untreated) genes in OM3 with expression ratio ≥2 and a *p*-value threshold < 0.05

| b number | GeneSymbol | | Function | | *p*-value | | Log_2_ Fold- Change^#^ |
| --- | --- | --- | --- | --- | --- | --- | --- |
| b4036 | *lamB* | | phage lambda receptor protein; maltose high-affinity receptor | | 0.039 | | -8.997 |
| b4240 | *treB* | | PTS system enzyme II, trehalose specific | | 0.042 | | -9.071 |
| b1966 | *tnaA* | | tryptophanase | | 0.049 | | -9.058 |
|  | | |  |  | |  | |
| b4034 | *malE* | | periplasmic maltose-binding protein; substrate recognition for transport and chemotaxis | | 0.043 | | -8.930 |
| b4239 | *treC* | | trehalase 6-P hydrolase | | 0.021 | | -8.536 |
| b4035 | *malK* | | ATP-binding component of transport system for maltose | | 0.039 | | -8.502 |
| b3115 | *tdcD* | | putative kinase | | 0.047 | | -8.075 |
| b3118 | *tdcA* | | transcriptional activator of *tdc* operon | | 0.039 | | -8.020 |
| b2149 | *mglA* | | ATP-binding component of methyl-galactoside transport and galactose taxis | | 0.044 | | -7.653 |
| b4033 | *malF* | | part of maltose permease, periplasmic | | 0.044 | | -7.592 |
| b2150 | *mglB* | | galactose-binding transport protein; receptor for galactose taxis | | 0.037 | | -7.230 |
| b3126 | *garL* | | orf, hypothetical protein | | 0.043 | | -6.819 |
| b4037 | *malM* | | periplasmic protein of mal regulon | | 0.040 | | -6.619 |
| b3709 | *tnaB* | | low affinity tryptophan permease | | 0.043 | | -6.596 |
| b3113 | *tdcF* | | orf, hypothetical protein | | 0.032 | | -6.399 |
| b3112 |  | | putative L-serine dehydratase | | 0.039 | | -6.340 |
| b2148 | *mglC* | | methyl-galactoside transport and galactose taxis | | 0.037 | | -6.168 |
| b1498 | *ydeN* | | putative sulfatase | | 0.043 | | -6.125 |
|  | |  | | | | | |
| b4032 | *malG* | | part of maltose permease, inner membrane | | 0.021 | | -5.942 |
| b3128 | *garD* | | putative hydrolase | | 0.039 | | -5.720 |
| b4119 | *melA* | | alpha-galactosidase | | 0.039 | | -5.703 |
| b3111 |  | | putative L-serine dehydratase | | 0.036 | | -5.678 |
| b1073 | *flgB* | | flagellar biosynthesis, cell-proximal portion of basal-body rod | | 0.021 | | -5.635 |
| b3125 | *garR* | | putative dehydrogenase | | 0.039 | | -5.590 |
| b1074 | *flgC* | | flagellar biosynthesis, cell-proximal portion of basal-body rod | | 0.039 | | -5.370 |
| b3225 | *nanA* | | N-acetylneuraminate lyase | | 0.042 | | -5.240 |
| b1075 | *flgD* | | flagellar biosynthesis, initiation of hook assembly | | 0.037 | | -5.225 |
| b4189 | *yjfO* | | orf, hypothetical protein | | 0.042 | | -5.047 |
| b2704 | *srlB* | | PTS system, glucitol/sorbitol-specific enzyme IIA component | | 0.047 | | -5.031 |
| b1076 | *flgE* | | flagellar biosynthesis, hook protein | | 0.037 | | -4.963 |
| b2000 | *flu* | | outer membrane fluffing protein, similar to adhesin | | 0.021 | | -4.879 |
| b3124 | *garK* | | orf, hypothetical protein | | 0.044 | | -4.836 |
| b3926 | *glpK* | | glycerol kinase | | 0.041 | | -4.733 |
| b3927 | *glpF* | | facilitated diffusion of glycerol | | 0.044 | | -4.717 |
| b4189 | *yjfO* | | orf, hypothetical protein | | 0.040 | | -4.660 |
| b1922 | *fliA* | | flagellar biosynthesis; alternative sigma factor 28; regulation of flagellar operons | | 0.027 | | -4.655 |
| b3528 | *dctA* | | uptake of C4-dicarboxylic acids | | 0.039 | | -4.617 |
| b2789 | *gudP* | | putative transport protein | | 0.039 | | -4.572 |
| b1493 | *gadB* | | glutamate decarboxylase isozyme | | 0.039 | | 4.571 |
| b3566 | *xylF* | | xylose binding protein transport system | | 0.047 | | -4.564 |
| b3517 | *gadA* | | glutamate decarboxylase isozyme | | 0.044 | | 4.517 |
| b4188 | *yjfN* | | orf, hypothetical protein | | 0.039 | | -4.445 |
| b3666 | *uhpT* | | hexose phosphate transport protein | | 0.042 | | -4.398 |
| b0929 | *ompF* | | outer membrane protein 1a | | 0.037 | | -4.312 |
| b1963 | *yedR* | | orf, hypothetical protein | | 0.039 | | 4.255 |
| b1945 | *fliM* | | flagellar biosynthesis, component of motor switch and energizing, enabling rotation and determining its direction | | 0.021 | | -4.246 |
| b1077 | *flgF* | | flagellar biosynthesis, cell-proximal portion of basal-body rod | | 0.027 | | -4.060 |
| b4299 | *yjhI* | | putative regulator | | 0.039 | | -4.031 |
| b2001 | *yeeR* | | orf, hypothetical protein | | 0.040 | | -4.010 |
| b4085 | *alsE* | | putative epimerase | | 0.040 | | -3.961 |
| b3133 | *agaV* | | PTS system, cytoplasmic, N-acetylgalactosamine-specific IIB component 2 | | 0.021 | | -3.961 |
| b0485 | *ybaS* | | putative glutaminase | | 0.039 | | 3.942 |
| b1944 | *fliL* | | flagellar biosynthesis | | 0.039 | | -3.935 |
| b1946 | *fliN* | | flagellar biosynthesis, component of motor switch and energizing, enabling rotation and determining its direction | | 0.042 | | -3.913 |
| b2801 | *fucP* | | fucose permease | | 0.043 | | -3.870 |
| b2788 | *gudX* | | putative glucarate dehydratase | | 0.037 | | -3.807 |
| b1497 | *ydeM* | | putative enzyme | | 0.039 | | -3.790 |
| b1005 |  | | orf, hypothetical protein | | 0.039 | | 3.773 |
| b4055 | *aphA* | | diadenosine tetraphosphatase | | 0.039 | | -3.767 |
| b4084 | *alsK* | | putative NAGC-like transcriptional regulator | | 0.039 | | -3.762 |
| b1078 | *flgG* | | flagellar biosynthesis, cell-distal portion of basal-body rod | | 0.027 | | -3.710 |
| b4307 | *yjhQ* | | orf, hypothetical protein | | 0.043 | | -3.689 |
| b1079 | *flgH* | | flagellar biosynthesis, basal-body outer-membrane L | | 0.037 | | -3.659 |
| b1938 | *fliF* | | flagellar biosynthesis; basal-body MS | | 0.039 | | -3.655 |
| b1901 | *araF* | | L-arabinose-binding periplasmic protein | | 0.039 | | -3.634 |
| b3946 | *fsaB* | | putative transaldolase | | 0.040 | | -3.628 |
| b4120 | *melB* | | melibiose permease II | | 0.039 | | -3.605 |
| b4086 | *alsC* | | putative transport system permease protein | | 0.038 | | -3.548 |
| b3020 | *ygiS* | | putative transport periplasmic protein | | 0.042 | | -3.457 |
| b3571 | *malS* | | alpha-amylase | | 0.021 | | -3.449 |
|  |  | | partial putative periplasmic transport protein Z2474 | | 0.039 | | -3.443 |
| b2799 | *fucO* | | L-1,2-propanediol oxidoreductase | | 0.046 | | -3.442 |
| b0651 | *rihA* | | putative tRNA synthetase | | 0.042 | | -3.381 |
| b2219 | *atoS* | | sensor protein AtoS for response regulator AtoC | | 0.039 | | -3.324 |
| b3092 | *uxaC* | | uronate isomerase | | 0.043 | | -3.309 |
| b1080 | *flgI* | | homolog of Salmonella P-ring of flagella basal body | | 0.040 | | -3.308 |
| b4322 | *uxuA* | | mannonate hydrolase | | 0.036 | | -3.275 |
| b1776 | *ydjL* | | putative oxidoreductase | | 0.039 | | -3.266 |
| b3417 | *malP* | | maltodextrin phosphorylase | | 0.037 | | -3.261 |
| b2800 | *fucA* | | L-fuculose-1-phosphate aldolase | | 0.045 | | -3.256 |
| b3223 | *nanE* | | putative enzyme | | 0.044 | | -3.183 |
| b1940 | *fliH* | | flagellar biosynthesis; export of flagellar proteins? | | 0.045 | | -3.156 |
| b4321 | *gntP* | | gluconate transport system permease 3 | | 0.027 | | -3.150 |
| b2924 | *mscS* | | putative transport protein | | 0.047 | | 3.127 |
| b3418 | *malT* | | positive regulator of mal regulon | | 0.039 | | -3.102 |
| b1521 | *uxaB* | | altronate oxidoreductase | | 0.044 | | -3.100 |
| b1947 | *fliO* | | flagellar biosynthesis | | 0.043 | | -2.933 |
| b2869 | *ygeV* | | putative transcriptional regulator | | 0.037 | | -2.877 |
| b3091 | *uxaA* | | altronate hydrolase | | 0.042 | | -2.849 |
| b2220 | *atoC* | | response regulator of ato, ornithine decarboxylase antizyme | | 0.037 | | -2.820 |
| b3949 | *frwC* | | PTS system, fructose-like enzyme II component | | 0.047 | | -2.807 |
| b1921 | *fliZ* | | orf, hypothetical protein | | 0.037 | | -2.788 |
| b1976 | *mtfA* | | orf, hypothetical protein | | 0.040 | | -2.784 |
| b3087 | *ygjR* | | orf, hypothetical protein | | 0.039 | | -2.766 |
| b4352 | *yjiA* | | orf, hypothetical protein | | 0.044 | | -2.745 |
| b2272 | *yfbM* | | orf, hypothetical protein | | 0.021 | | -2.742 |
| b3135 | *agaA* | | putative N-acetylgalactosamine-6-phosphate deacetylase | | 0.041 | | -2.740 |
| b1897 | *otsB* | | trehalose-6-phosphate phophatase, biosynthetic | | 0.037 | | 2.717 |
| b1732 | *katE* | | catalase; hydroperoxidase HPII | | 0.027 | | 2.701 |
| b0871 | *poxB* | | pyruvate oxidase | | 0.021 | | 2.696 |
| b2238 | *yfaH* | | orf, hypothetical protein | | 0.021 | | -2.686 |
| b1795 | *yeaQ* | | orf, hypothetical protein | | 0.037 | | 2.653 |
| b4216 | *ytfJ* | | orf, hypothetical protein | | 0.041 | | -2.639 |
| b2464 | *talA* | | transaldolase A | | 0.047 | | 2.627 |
| b4568 | *ytjA* | | predicted protein | | 0.039 | | 2.594 |
| b1311 | *ycjO* | | putative binding-protein dependent transport protein | | 0.037 | | -2.592 |
| b1896 | *otsA* | | trehalose-6-phosphate synthase | | 0.037 | | 2.581 |
| b1900 | *araG* | | ATP-binding component of high-affinity L-arabinose transport system | | 0.037 | | -2.580 |
| b3748 | *rbsD* | | D-ribose high-affinity transport system; membrane-associated protein | | 0.039 | | -2.525 |
| b4003 | *zraS* | | sensor kinase for HydG, hydrogenase 3 activity | | 0.043 | | -2.518 |
| b3516 | *gadX* | | putative ARAC-type regulatory protein | | 0.046 | | 2.483 |
| b0897 | *ycaC* | | orf, hypothetical protein | | 0.044 | | 2.482 |
|  | *ybdD* | | conserved protein b4512 | | 0.043 | | -2.431 |
| b4089 | *rpiR* | | transcriptional repressor of rpiB expression | | 0.043 | | -2.401 |
| b1171 | *ymgD* | | orf, hypothetical protein | | 0.048 | | 2.392 |
| b1518 | *lsrG* | | orf, hypothetical protein | | 0.048 | | -2.380 |
| b3263 | *yhdU* | | orf, hypothetical protein | | 0.040 | | -2.379 |
| b4193 | *ulaA* | | orf, hypothetical protein | | 0.039 | | -2.354 |
| b1308 | *pspE* | | phage shock protein | | 0.036 | | -2.352 |
| b3371 | *frlB* | | putative transport protein | | 0.037 | | -2.344 |
| b4353 | *yjiX* | | orf, hypothetical protein | | 0.039 | | -2.330 |
| b1072 | *flgA* | | flagellar biosynthesis; assembly of basal-body periplasmic P ring | | 0.037 | | -2.271 |
| b2465 | *tktB* | | transketolase 2 isozyme | | 0.042 | | 2.259 |
| b3426 | *glpD* | | *sn*-glycerol-3-phosphate dehydrogenase | | 0.050 | | -2.237 |
| b1384 | *feaR* | | regulatory protein for 2-phenylethylamine catabolism | | 0.039 | | -2.230 |
| b1081 | *flgJ* | | flagellar biosynthesis | | 0.039 | | -2.220 |
| b0044 | *fixX* | | putative ferredoxin | | 0.046 | | -2.218 |
| b2341 | *fadJ* | | putative enzyme | | 0.039 | | -2.188 |
| b3515 | *gadW* | | putative ARAC-type regulatory protein | | 0.042 | | 2.170 |
| b2366 | *dsdA* | | D-serine dehydratase | | 0.039 | | -2.110 |
| b3357 | *crp* | | cyclic AMP receptor protein | | 0.027 | | 2.067 |
| b3544 | *dppA* | | dipeptide transport protein | | 0.024 | | -2.065 |
| b3880 | *yihS* | | orf, hypothetical protein | | 0.044 | | -2.031 |
| b4090 | *rpiB* | | ribose 5-phosphate isomerase B | | 0.048 | | -2.022 |
| b3239 | *yhcO* | | orf, hypothetical protein | | 0.047 | | 2.022 |
| b0453 | *ybaY* | | glycoprotein/polysaccharide metabolism | | 0.039 | | 2.021 |
| b0061 | *araD* | | L-ribulose-5-phosphate 4-epimerase | | 0.039 | | -2.012 |

^#^ - Logarithmic (base 2) value of expression ratio of genes in OM3 compared to WT without H_2_O_2_ treatment
