# Supplementary material for: Enhancing E. coli Tolerance towards Oxidative Stress via Engineering Its Global Regulator cAMP Receptor Protein (CRP)
Source: PLoS One. 2012 Dec 14;7(12):e51179. doi: 10.1371/journal.pone.0051179 (PMC3522674; doi:10.1371/journal.pone.0051179)
Supplement: Table S2 — Genes in OM3 with expression ratio ≥2 and a p-value threshold <0.05 after H2O2 treatment. (DOC) [file pone.0051179.s009.doc]

**TABLE S2** Genes in OM3 with expression ratio ≥ 2 and a *p*-value threshold < 0.05 after H_2_O_2_ treatment

| b number | GeneSymbol | Function | *p*-value | Log_2_Fold- Change^*^ |
| --- | --- | --- | --- | --- |
| b3517 | *gadA* | glutamate decarboxylase isozyme | 0.024 | 7.766 |
| b1493 | *gadB* | glutamate decarboxylase isozyme | 0.033 | 7.097 |
| b1492 | *gadC* | acid sensitivity protein, putative transporter | 0.031 | 7.025 |
| b2850 | *ygeF* | orf, hypothetical protein | 0.024 | 6.323 |
| b1005 |  | orf, hypothetical protein | 0.024 | 6.199 |
| b3117 | *tdcB* | threonine dehydratase, catabolic | 0.046 | -6.162 |
| b3566 | *xylF* | xylose binding protein transport system | 0.024 | -6.078 |
| b3491 | *yhiM* | orf, hypothetical protein | 0.028 | 5.820 |
| b1259 | *yciG* | orf, hypothetical protein | 0.031 | 5.757 |
| b3115 | *tdcD* | putative kinase | 0.029 | -5.722 |
| b3116 | *tdcC* | anaerobically inducible L-threonine, L-serine permease | 0.025 | -5.632 |
| b3708 | *tnaA* | tryptophanase | 0.024 | -5.501 |
| b3118 | *tdcA* | transcriptional activator of *tdc* operon | 0.028 | -5.493 |
| b0929 | *ompF* | outer membrane protein 1a | 0.024 | -5.363 |
| b4307 | *yjhQ* | orf, hypothetical protein | 0.025 | -5.133 |
| b0598 | *cstA* | carbon starvation protein | 0.027 | -5.093 |
| b4240 | *treB* | PTS system enzyme II, trehalose specific | 0.025 | -5.075 |
| b4518 | *ymdF* | conserved protein | 0.025 | 5.003 |
| b3511 | *hdeD* | orf, hypothetical protein | 0.024 | 4.967 |
| b2844 | *yqeF* | putative acyltransferase | 0.028 | -4.791 |
| b4036 | *lamB* | phage lambda receptor protein; maltose high-affinity receptor | 0.024 | -4.780 |
| b3081 | *fadH* | putative NADPH dehydrogenase | 0.025 | -4.761 |
| b2342 | *fadI* | putative acyltransferase | 0.036 | -4.695 |
| b1074 | *flgC* | flagellar biosynthesis, cell-proximal portion of basal-body rod | 0.028 | -4.692 |
| b2150 | *mglB* | galactose-binding transport protein; receptor for galactose taxis | 0.025 | -4.616 |
| b4566 | *yjhX* | conserved protein | 0.033 | -4.615 |
| b1073 | *flgB* | flagellar biosynthesis, cell-proximal portion of basal-body rod | 0.031 | -4.592 |
| b0978 | *appC* | probable third cytochrome oxidase, subunit I | 0.024 | 4.592 |
| b2801 | *fucP* | fucose permease | 0.028 | -4.532 |
| b3567 | *xylG* | putative ATP-binding protein of xylose transport system | 0.028 | -4.484 |
| b3509 | *hdeB* | orf, hypothetical protein | 0.024 | 4.458 |
| b1422 | *ydcI* | putative transcriptional regulator LYSR-type | 0.040 | -4.457 |
| b4055 | *aphA* | diadenosine tetraphosphatase | 0.039 | -4.340 |
| b2957 | *ansB* | periplasmic L-asparaginase II | 0.024 | -4.338 |
| b3846 | *fadB* | 4-enzyme protein: 3-hydroxyacyl-CoA dehydrogenase; 3-hydroxybutyryl-CoA epimerase; delta | 0.032 | -4.294 |
| b1075 | *flgD* | flagellar biosynthesis, initiation of hook assembly | 0.024 | -4.266 |
| b4034 | *malE* | periplasmic maltose-binding protein; substrate recognition for transport and chemotaxis | 0.032 | -4.257 |
| b0753 | *ybgS* | putative homeobox protein | 0.024 | 4.238 |
| b2537 | *hcaR* | transcriptional activator of *hca* cluster | 0.046 | -4.234 |
| b0722 | *sdhD* | succinate dehydrogenase, hydrophobic subunit | 0.024 | -4.214 |
| b4187 | *aidB* | putative acyl coenzyme A dehydrogenase | 0.028 | 4.197 |
| b1256 | *ompW* | putative outer membrane protein | 0.024 | -4.196 |
| b2802 | *fucI* | L-fucose isomerase | 0.032 | -4.177 |
| b3364 | *tsgA* | putative transport | 0.025 | 4.166 |
| b1497 | *ydeM* | putative enzyme | 0.027 | -4.162 |
| b4069 | *acs* | acetyl-CoA synthetase | 0.024 | -4.143 |
| b0721 | *sdhC* | succinate dehydrogenase, cytochrome | 0.022 | -4.139 |
| b1536 | *ydeI* | orf, hypothetical protein | 0.028 | 4.125 |
| b0622 | *pagP* | orf, hypothetical protein | 0.029 | 4.109 |
| b1137 | *ymfD* | orf, hypothetical protein | 0.033 | -4.105 |
| b1963 | *yedR* | orf, hypothetical protein | 0.029 | 4.098 |
| b3528 | *dctA* | uptake of C4-dicarboxylic acids | 0.027 | -4.086 |
| b0979 | *appB* | probable third cytochrome oxidase, subunit II | 0.024 | 4.077 |
| b2135 | *yohC* | orf, hypothetical protein | 0.024 | 4.046 |
| b3510 | *hdeA* | orf, hypothetical protein | 0.024 | 4.020 |
| b4189 | *yjfO* | orf, hypothetical protein | 0.040 | -3.998 |
| b4068 | *yjcH* | orf, hypothetical protein | 0.024 | -3.963 |
| b0972 | *hyaA* | hydrogenase-1 small subunit | 0.033 | 3.961 |
| b2924 | *mscS* | putative transport protein | 0.037 | 3.897 |
| b2241 | *glpA* | *sn*-glycerol-3-phosphate dehydrogenase | 0.033 | -3.864 |
| b0806 | *ybiM* | orf, hypothetical protein | 0.028 | 3.846 |
| b3512 | *gadE* | orf, hypothetical protein | 0.031 | 3.821 |
| b1138 | *ymfE* | orf, hypothetical protein | 0.030 | -3.811 |
| b1732 | *katE* | catalase; hydroperoxidase HPII | 0.035 | 3.801 |
| b2980 | *glcC* | transcriptional activator for glc operon | 0.045 | -3.772 |
| b3749 | *rbsA* | ATP-binding component of D-ribose high-affinity transport system | 0.031 | -3.768 |
| b4030 | *psiE* | orf, hypothetical protein | 0.026 | 3.767 |
| b4299 | *yjhI* | putative regulator | 0.032 | -3.718 |
| b0485 | *ybaS* | putative glutaminase | 0.031 | 3.707 |
| b3092 | *uxaC* | uronate isomerase | 0.034 | -3.690 |
| b2172 | *yeiQ* | putative oxidoreductase | 0.024 | -3.681 |
| b2149 | *mglA* | ATP-binding component of methyl-galactoside transport and galactose taxis | 0.040 | -3.660 |
| b0806 | *ybiM* | orf, hypothetical protein | 0.046 | 3.635 |
| b2165 | *yeiN* | orf, hypothetical protein | 0.024 | -3.632 |
| b1612 | *fumA* | fumarase A = fumarate hydratase Class I; aerobic isozyme | 0.028 | -3.622 |
| b0486 | *ybaT* | putative amino acid/amine transport protein | 0.036 | 3.620 |
| b3927 | *glpF* | facilitated diffusion of glycerol | 0.028 | -3.619 |
| b2464 | *talA* | transaldolase A | 0.026 | 3.608 |
| b4216 | *ytfJ* | orf, hypothetical protein | 0.024 | -3.599 |
| b1685 | *ydiH* | orf, hypothetical protein | 0.024 | 3.599 |
| b2151 | *galS* | mgl repressor, galactose operon inducer | 0.031 | -3.572 |
| b2240 | *glpT* | *sn*-glycerol-3-phosphate permease | 0.028 | -3.561 |
| b2206 | *napA* | probable nitrate reductase 3 | 0.040 | -3.558 |
| b0871 | *poxB* | pyruvate oxidase | 0.024 | 3.536 |
| b4239 | *treC* | trehalase 6-P hydrolase | 0.030 | -3.516 |
| b3006 | *exbB* | uptake of enterochelin; tonB-dependent uptake of *B. colicins* | 0.024 | 3.515 |
| b1076 | *flgE* | flagellar biosynthesis, hook protein | 0.027 | -3.513 |
| b1015 | *putP* | major sodium/proline symporter | 0.024 | -3.504 |
| b1683 | *sufB* | orf, hypothetical protein | 0.044 | 3.483 |
| b1528 | *ydeA* | putative resistance / regulatory protein | 0.043 | 3.477 |
| b4189 | *yjfO* | orf, hypothetical protein | 0.041 | -3.474 |
| b1967 | *hchA* | orf, hypothetical protein | 0.024 | 3.469 |
| b1415 | *aldA* | aldehyde dehydrogenase, NAD-linked | 0.033 | -3.467 |
| b3241 | *aaeA* | putative membrane protein | 0.044 | 3.454 |
| b3513 | *mdtE* | putative membrane protein | 0.040 | 3.442 |
| b1922 | *fliA* | flagellar biosynthesis; alternative sigma factor 28; regulation of flagellar operons | 0.026 | -3.439 |
| b2766 | *ygcN* | orf, hypothetical protein | 0.044 | -3.431 |
| b3091 | *uxaA* | altronate hydrolase | 0.024 | -3.426 |
| b2876 | *yqeC* | orf, hypothetical protein | 0.039 | -3.423 |
| b3506 | *slp* | outer membrane protein induced after carbon starvation | 0.033 | 3.423 |
| b2166 | *yeiC* | putative kinase | 0.026 | -3.405 |
| b3753 | *rbsR* | regulator for rbs operon | 0.044 | -3.398 |
| b4067 | *actP* | putative transport protein | 0.030 | -3.396 |
| b3555 | *yiaG* | orf, hypothetical protein | 0.038 | 3.386 |
| b3113 | *tdcF* | orf, hypothetical protein | 0.024 | -3.382 |
| b2997 | *hybO* | putative hydrogenase subunit | 0.024 | -3.374 |
| b2239 | *glpQ* | glycerophosphodiester phosphodiesterase, periplasmic | 0.044 | -3.372 |
| b4367 | *fhuF* | orf, hypothetical protein | 0.024 | 3.371 |
| b1737 | *chbC* | PEP-dependent phosphotransferase enzyme II for cellobiose, arbutin, and salicin | 0.024 | -3.370 |
| b0848 | *ybjM* | orf, hypothetical protein | 0.028 | 3.364 |
| b4199 | *yjfY* | orf, hypothetical protein | 0.025 | 3.358 |
| b1478 | *adhP* | alcohol dehydrogenase | 0.024 | 3.355 |
| b3926 | *glpK* | glycerol kinase | 0.038 | -3.343 |
| b3132 | *kbaZ* | putative tagatose 6-phosphate kinase 2 | 0.028 | -3.337 |
| b1684 | *sufA* | orf, hypothetical protein | 0.024 | 3.336 |
| b2767 | *ygcO* | orf, hypothetical protein | 0.036 | -3.335 |
| b3752 | *rbsK* | ribokinase | 0.024 | -3.314 |
| b3750 | *rbsC* | D-ribose high-affinity transport system | 0.028 | -3.276 |
| b2869 | *ygeV* | putative transcriptional regulator | 0.033 | -3.273 |
| b4188 | *yjfN* | orf, hypothetical protein | 0.035 | -3.265 |
| b2996 | *hybA* | hydrogenase-2 small subunit | 0.024 | -3.245 |
| b0342 | *lacA* | thiogalactoside acetyltransferase | 0.024 | -3.244 |
| b1930 | *yedF* | orf, hypothetical protein | 0.024 | -3.235 |
| b1901 | *araF* | L-arabinose-binding periplasmic protein | 0.050 | -3.228 |
| b4298 | *yjhH* | putative lyase/synthase | 0.024 | -3.210 |
| b4342 | *yjiT* | orf, hypothetical protein | 0.024 | -3.170 |
| b1467 | *narY* | cryptic nitrate reductase 2, beta subunit | 0.024 | 3.169 |
| b1681 | *sufD* | orf, hypothetical protein | 0.043 | 3.140 |
| b0681 | *ybfM* | orf, hypothetical protein | 0.031 | -3.130 |
| b3005 | *exbD* | uptake of enterochelin; tonB-dependent uptake of *B. colicins* | 0.028 | 3.110 |
| b1309 | *ycjM* | putative polysaccharide hydrolase | 0.041 | -3.099 |
| b4060 | *yjcB* | orf, hypothetical protein | 0.031 | 3.077 |
| b2220 | *atoC* | response regulator of ato, ornithine decarboxylase antizyme | 0.025 | -3.072 |
| b0897 | *ycaC* | orf, hypothetical protein | 0.034 | 3.071 |
| b1466 | *narW* | cryptic nitrate reductase 2, delta subunit, assembly function | 0.024 | 3.070 |
| b0594 | *entE* | 2,3-dihydroxybenzoate-AMP ligase | 0.026 | 3.067 |
| b4376 | *osmY* | hyperosmotically inducible periplasmic protein | 0.024 | 3.062 |
| b1258 | *yciF* | putative structural proteins | 0.033 | 3.058 |
| b1537 | *ydeJ* | orf, hypothetical protein | 0.037 | 3.056 |
| b0030 | *rihC* | orf, hypothetical protein | 0.024 | -3.054 |
| b1661 | *cfa* | cyclopropane fatty acyl phospholipid synthase | 0.024 | 3.046 |
| b2243 | *glpC* | *sn*-glycerol-3-phosphate dehydrogenase | 0.025 | -3.040 |
| b4323 | *uxuB* | D-mannonate oxidoreductase | 0.034 | -3.037 |
| b1945 | *fliM* | flagellar biosynthesis, component of motor switch and energizing, enabling rotation and determining its direction | 0.033 | -3.036 |
| b1795 | *yeaQ* | orf, hypothetical protein | 0.047 | 3.023 |
| b1897 | *otsB* | trehalose-6-phosphate phophatase, biosynthetic | 0.035 | 3.014 |
| b3873 | *yihM* | orf, hypothetical protein | 0.032 | -3.001 |
| b0980 | *appA* | phosphoanhydride phosphorylase; pH 2.5 acid phosphatase; periplasmic | 0.025 | 3.000 |
| b1896 | *otsA* | trehalose-6-phosphate synthase | 0.012 | 2.996 |
| b3073 | *ygjG* | probable ornithine aminotransferase | 0.024 | 2.969 |
| b3748 | *rbsD* | D-ribose high-affinity transport system; membrane-associated protein | 0.025 | -2.961 |
| b2462 | *eutS* | orf, hypothetical protein | 0.039 | -2.959 |
| b3112 |  | putative L-serine dehydratase | 0.024 | -2.955 |
| b0723 | *sdhA* | succinate dehydrogenase, flavoprotein subunit | 0.028 | -2.929 |
| b2310 | *argT* | lysine-, arginine-, ornithine-binding periplasmic protein | 0.035 | -2.901 |
| b4227 | *ytfQ* | putative LACI-type transcriptional regulator | 0.024 | -2.889 |
| b0974 | *hyaC* | probable Ni/Fe-hydrogenase 1 b-type cytochrome subunit | 0.026 | 2.889 |
| b4346 | *mcrB* | component of McrBC 5-methylcytosine restriction system | 0.035 | -2.883 |
| b2460 | *eutQ* | orf, hypothetical protein | 0.031 | -2.881 |
| b1252 | *tonB* | energy transducer; uptake of iron, cyanocobalimin; sensitivity to phages, colicins | 0.043 | 2.877 |
| b1465 | *narV* | cryptic nitrate reductase 2, gamma subunit | 0.024 | 2.874 |
| b3240 | *aaeB* | orf, hypothetical protein | 0.022 | 2.856 |
| b4293 | *fecI* | probable RNA polymerase sigma factor | 0.037 | 2.851 |
| b3153 | *yhbO* | orf, hypothetical protein | 0.024 | 2.840 |
| b2426 | *ucpA* | putative oxidoreductase | 0.033 | -2.835 |
| b0899 | *ycaM* | putative transport | 0.024 | -2.834 |
| b3133 | *agaV* | PTS system, cytoplasmic, N-acetylgalactosamine-specific IIB component 2 | 0.024 | -2.823 |
| b4511 | *ybdZ* | conserved protein | 0.042 | 2.814 |
| b1778 | *msrB* | orf, hypothetical protein | 0.038 | -2.812 |
| b2080 | *yegP* | orf, hypothetical protein | 0.022 | 2.798 |
| b0596 | *entA* | 2,3-dihydro-2,3-dihydroxybenzoate dehydrogenase, enterochelin biosynthesis | 0.024 | 2.796 |
| b4107 | *yjdN* | orf, hypothetical protein | 0.033 | 2.792 |
| b1892 | *flhD* | regulator of flagellar biosynthesis, acting on class 2 operons; transcriptional initiation factor | 0.037 | -2.790 |
| b2204 | *napH* | ferredoxin-type protein: electron transfer | 0.036 | -2.780 |
| b1195 | *ymgE* | orf, hypothetical protein | 0.024 | 2.778 |
| b4269 | *yjgB* | putative oxidoreductase | 0.024 | 2.775 |
| b2148 | *mglC* | methyl-galactoside transport and galactose taxis | 0.024 | -2.772 |
| b1680 | *sufS* | orf, hypothetical protein | 0.024 | 2.771 |
| b2344 | *fadL* | transport of long-chain fatty acids; sensitivity to phage T2 | 0.031 | -2.763 |
| b0790 | *ybhP* | orf, hypothetical protein | 0.025 | 2.763 |
| b1482 | *osmC* | osmotically inducible protein | 0.038 | 2.755 |
| b0802 | *ybiJ* | orf, hypothetical protein | 0.037 | 2.746 |
| b1891 | *flhC* | regulator of flagellar biosynthesis acting on class 2 operons; transcription initiation factor | 0.032 | -2.743 |
| b0789 | *ybhO* | putative synthetase | 0.044 | 2.742 |
|  | *Z0666* | orf Unknown function Z0666 | 0.035 | -2.736 |
| b1241 | *adhE* | CoA-linked acetaldehyde dehydrogenase and iron-dependent alcohol dehydrogenase; pyruvate-formate-lyase deactivase | 0.024 | 2.735 |
| b1905 | *ftnA* | cytoplasmic ferritin | 0.040 | -2.728 |
| b2799 | *fucO* | L-1,2-propanediol oxidoreductase | 0.029 | -2.723 |
| b0720 | *gltA* | citrate synthase | 0.034 | -2.697 |
| b3365 | *nirB* | nitrite reductase | 0.030 | 2.688 |
| b4321 | *gntP* | gluconate transport system permease 3 | 0.040 | -2.681 |
| b3925 | *glpX* | unknown function in glycerol metabolism | 0.026 | -2.675 |
| b1329 | *mppA* | putative transport periplasmic protein | 0.026 | -2.633 |
| b0343 | *lacY* | galactoside permease | 0.024 | -2.630 |
| b0651 | *rihA* | putative tRNA synthetase | 0.028 | -2.622 |
| b1000 | *cbpA* | curved DNA-binding protein; functions closely related to DnaJ | 0.032 | 2.610 |
| b0384 | *psiF* | induced by phosphate starvation | 0.035 | 2.609 |
| b4122 | *fumB* | fumarase B= fumarate hydratase Class I; anaerobic isozyme | 0.028 | -2.601 |
| b3507 | *dctR* | orf, hypothetical protein | 0.041 | 2.585 |
| b0999 | *cbpM* | orf, hypothetical protein | 0.024 | 2.583 |
| b3077 | *ebgC* | evolved beta-D-galactosidase, beta subunit; cryptic gene | 0.041 | -2.581 |
| b1218 | *chaC* | cation transport regulator | 0.042 | 2.577 |
| b2672 | *ygaM* | orf, hypothetical protein | 0.033 | 2.556 |
| b1679 | *sufE* | orf, hypothetical protein | 0.032 | 2.551 |
| b2347 | *yfdC* | putative transport | 0.033 | 2.540 |
| b3909 | *kdgT* | 2-keto-3-deoxy-D-gluconate transport system | 0.026 | -2.529 |
| b0585 | *fes* | enterochelin esterase | 0.024 | 2.515 |
| b3134 | *agaW* | PTS system N-acetylgalactosameine-specific IIC component 2 | 0.024 | -2.505 |
| b1751 | *ydjY* | orf, hypothetical protein | 0.043 | -2.502 |
| b3946 | *fsaB* | putative transaldolase | 0.024 | -2.494 |
| b0975 | *hyaD* | processing of HyaA and HyaB proteins | 0.024 | 2.493 |
| b1036 | *ycdZ* | orf, hypothetical protein | 0.025 | 2.480 |
| b1770 | *ydjF* | putative DEOR-type transcriptional regulator | 0.033 | -2.480 |
| b1927 | *amyA* | cytoplasmic alpha-amylase | 0.026 | 2.479 |
| b3366 | *nirD* | nitrite reductase | 0.024 | 2.478 |
| b2687 | *luxS* | orf, hypothetical protein | 0.037 | 2.467 |
| b2665 | *ygaU* | orf, hypothetical protein | 0.028 | 2.455 |
| b2943 | *galP* | galactose-proton symport of transport system | 0.025 | 2.451 |
| b1838 | *pphA* | protein phosphatase 1 modulates phosphoproteins, signals protein misfolding | 0.032 | 2.447 |
| b4045 | *yjbJ* | orf, hypothetical protein | 0.049 | 2.446 |
| b4292 | *fecR* | regulator for fec operon, periplasmic | 0.030 | 2.435 |
| b2302 | *yfcG* | putative S-transferase | 0.024 | 2.429 |
| b4133 | *cadC* | transcriptional activator of cad operon | 0.038 | 2.425 |
| b2870 | *ygeW* | putative carbamoyl transferase | 0.024 | -2.425 |
| b3546 | *eptB* | orf, hypothetical protein | 0.029 | -2.416 |
| b2203 | *napB* | cytochrome c-type protein | 0.029 | -2.412 |
| b1189 | *dadA* | D-amino acid dehydrogenase subunit | 0.028 | -2.408 |
| b2244 | *yfaD* | orf, hypothetical protein | 0.028 | -2.408 |
| b1946 | *fliN* | flagellar biosynthesis, component of motor switch and energizing, enabling rotation and determining its direction | 0.027 | -2.393 |
| b1320 | *ycjW* | putative LACI-type transcriptional regulator | 0.045 | -2.387 |
| b1805 | *fadD* | acyl-CoA synthetase, long-chain-fatty-acid--CoA ligase | 0.040 | -2.377 |
| b1217 | *chaB* | cation transport regulator | 0.041 | 2.377 |
| b1678 | *ynhG* | orf, hypothetical protein | 0.028 | 2.368 |
| b2258 | *arnF* | putative transport/receptor protein | 0.040 | -2.362 |
| b3452 | *ugpA* | *sn*-glycerol 3-phosphate transport system, integral membrane protein | 0.035 | -2.356 |
| b3568 | *xylH* | putative xylose transport, membrane component | 0.026 | -2.356 |
| b1950 | *fliR* | flagellar biosynthesis | 0.024 | -2.352 |
| b3519 | *treF* | cytoplasmic trehalase | 0.033 | 2.347 |
| b3361 | *fic* | induced in stationary phase, recognized by rpoS, affects cell division | 0.028 | 2.342 |
| b4228 | *ytfR* | putative ATP-binding component of a transport system | 0.034 | -2.341 |
| b2800 | *fucA* | L-fuculose-1-phosphate aldolase | 0.027 | -2.340 |
| b2211 | *yojI* | putative ATP-binding component of a transport system | 0.038 | 2.319 |
| b3922 | *yiiS* | orf, hypothetical protein | 0.024 | 2.317 |
| b2463 | *maeB* | putative multimodular enzyme | 0.024 | -2.312 |
| b0724 | *sdhB* | succinate dehydrogenase, iron sulfur protein | 0.025 | -2.307 |
| b3418 | *malT* | positive regulator of mal regulon | 0.038 | -2.300 |
| b1190 | *dadX* | alanine racemase 2, catabolic | 0.034 | -2.296 |
| b0809 | *glnQ* | ATP-binding component of glutamine high-affinity transport system | 0.026 | -2.295 |
| b3362 | *yhfG* | orf, hypothetical protein | 0.037 | 2.294 |
| b4021 | *pepE* | peptidase E, a dipeptidase where amino-terminal residue is aspartate | 0.024 | -2.293 |
| b1938 | *fliF* | flagellar biosynthesis; basal-body MS | 0.031 | -2.287 |
| b2871 | *ygeX* | putative dehydratase | 0.038 | -2.284 |
| b3403 | *pck* | phosphoenolpyruvate carboxykinase | 0.033 | -2.279 |
| b0976 | *hyaE* | processing of HyaA and HyaB proteins | 0.035 | 2.277 |
| b2981 | *yghO* | orf, hypothetical protein | 0.031 | -2.264 |
| b2219 | *atoS* | sensor protein AtoS for response regulator AtoC | 0.026 | -2.260 |
| b4297 | *yjhG* | putative dehydratase | 0.042 | -2.258 |
| b3603 | *lldP* | L-lactate permease | 0.028 | -2.250 |
| b2242 | *glpB* | *sn*-glycerol-3-phosphate dehydrogenase | 0.024 | -2.244 |
| b4139 | *aspA* | aspartate ammonia-lyase | 0.024 | -2.236 |
| b4003 | *zraS* | sensor kinase for HydG, hydrogenase 3 activity | 0.041 | -2.235 |
| b0150 | *fhuA* | outer membrane protein receptor for ferrichrome, colicin M, and phages T1, T5, and phi80 | 0.026 | 2.234 |
| b0220 | *ivy* | orf, hypothetical protein | 0.041 | 2.232 |
| b1777 | *yeaC* | orf, hypothetical protein | 0.025 | -2.229 |
| b0380 | *yaiZ* | orf, hypothetical protein | 0.028 | -2.226 |
| b0592 | *fepB* | ferric enterobactin | 0.033 | 2.223 |
| b2341 | *fadJ* | putative enzyme | 0.038 | -2.221 |
| b2888 | *ygfU* | putative permease | 0.033 | -2.215 |
| b3514 | *mdtF* | putative transport system permease protein | 0.033 | 2.204 |
| b1646 | *sodC* | superoxide dismutase precursor | 0.031 | 2.195 |
| b3023 | *ygiV* | orf, hypothetical protein | 0.024 | 2.194 |
| b0810 | *glnP* | glutamine high-affinity transport system; membrane component | 0.033 | -2.193 |
| b3043 | *ygiL* | putative fimbrial-like protein | 0.046 | -2.178 |
| b2289 | *lrhA* | NADH dehydrogenase transcriptional regulator, LysR family | 0.032 | -2.172 |
| b4118 | *melR* | regulator of melibiose operon | 0.038 | -2.171 |
| b4229 |  | putative ATP-binding component of a transport system | 0.033 | -2.166 |
| b3020 | *ygiS* | putative transport periplasmic protein | 0.031 | -2.165 |
| b2878 | *ygfK* | putative oxidoreductase, Fe-S subunit | 0.041 | -2.165 |
| b0595 | *entB* | 2,3-dihydro-2,3-dihydroxybenzoate synthetase, isochroismatase | 0.046 | 2.163 |
| b4322 | *uxuA* | mannonate hydrolase | 0.036 | -2.163 |
| b0506 | *allR* | putative regulator | 0.046 | 2.161 |
| b1953 | *yodD* | orf, hypothetical protein | 0.030 | 2.159 |
| b2994 | *hybC* | probable large subunit, hydrogenase-2 | 0.046 | -2.155 |
| b2309 | *hisJ* | histidine-binding periplasmic protein of high-affinity histidine transport system | 0.037 | -2.152 |
| b1611 | *fumC* | fumarase C= fumarate hydratase Class II; isozyme | 0.025 | -2.147 |
| b0564 | *appY* | regulatory protein affecting appA and other genes | 0.024 | 2.142 |
| b4351 | *mrr* | restriction of methylated adenine | 0.035 | -2.126 |
| b1077 | *flgF* | flagellar biosynthesis, cell-proximal portion of basal-body rod | 0.024 | -2.114 |
| b2013 | *yeeE* | putative transport system permease protein | 0.048 | -2.114 |
| b1735 | *chbR* | negative transcriptional regulator of *cel* operon | 0.028 | -2.113 |
| b0118 | *acnB* | aconitate hydrase B | 0.033 | -2.104 |
| b2552 | *hmp* | dihydropteridine reductase, ferrisiderophore reductase activity | 0.035 | -2.092 |
| b3964 | *yijD* | orf, hypothetical protein | 0.041 | -2.077 |
| b3905 | *rhaS* | positive regulator for *rhaBAD* operon | 0.024 | -2.077 |
| b1750 | *ydjX* | orf, hypothetical protein | 0.028 | -2.075 |
| b0151 | *fhuC* | ATP-binding component of hydroxymate-dependent iron transport | 0.033 | 2.072 |
| b3544 | *dppA* | dipeptide transport protein | 0.047 | -2.070 |
| b0977 | *hyaF* | nickel incorporation into hydrogenase-1 proteins | 0.038 | 2.069 |
| b3917 | *sbp* | periplasmic sulfate-binding protein | 0.035 | -2.063 |
| b4037 | *malM* | periplasmic protein of mal regulon | 0.026 | -2.061 |
| b0624 | *crcB* | orf, hypothetical protein | 0.027 | 2.060 |
| b3070 | *yqjH* | orf, hypothetical protein | 0.032 | 2.059 |
| b0608 | *ybdR* | putative oxidoreductase | 0.037 | 2.059 |
| b1101 | *ptsG* | PTS system, glucose-specific IIBC component | 0.024 | 2.056 |
| b1504 | *ydeS* | putative fimbrial-like protein | 0.027 | -2.055 |
| b3004 |  | orf, hypothetical protein | 0.034 | 2.054 |
| b2415 | *ptsH* | PTS system protein HPr | 0.028 | 2.045 |
| b1078 | *flgG* | flagellar biosynthesis, cell-distal portion of basal-body rod | 0.035 | -2.039 |
| b2518 | *ndk* | nucleoside diphosphate kinase | 0.036 | -2.029 |
| b1676 | *pykF* | pyruvate kinase I | 0.028 | 2.026 |
| b2879 | *ssnA* | putative proteoglycan | 0.026 | -2.020 |
| b3923 | *uspD* | putative regulator | 0.025 | 2.019 |
| b1776 | *ydjL* | putative oxidoreductase | 0.033 | -2.005 |
| b2308 | *hisQ* | histidine transport system permease protein | 0.024 | -2.000 |

^*^ - Logarithmic (base 2) value of expression ratio of genes in OM3 compared to WT after treatment with 4 mM H_2_O_2_
