# Supplementary material for: Enhancing E. coli Tolerance towards Oxidative Stress via Engineering Its Global Regulator cAMP Receptor Protein (CRP)
Source: PLoS One. 2012 Dec 14;7(12):e51179. doi: 10.1371/journal.pone.0051179 (PMC3522674; doi:10.1371/journal.pone.0051179)
Supplement: Table S3 — qRT-PCR primers used in this study. (DOC) [file pone.0051179.s010.doc]

**TABLE S3** qRT-PCR primers used in this study^a^

| Primer |  | Sequence |
| --- | --- | --- |
|  |  |  |
|  |  |  |
| *crp_F* |  | TCAGAGAAAGTGGGCAACCTGG |
| *crp_R* |  | GATGGTTTTACCGTGTGCGGAG |
|  |  |  |
| *cya_F* |  | AGATTGATCAGGTGCGTGAGGC |
| *cya_R* |  | AAATCTGCGGGTTTACCAGCGT |
|  |  |  |
| *sodA*_F |  | GCTATCGAACGTGACTTCGGCT |
| *sodA*_R |  | TCAGCGGAGAATCCTGGTTAGC |
|  |  |  |
| *katE*_F |  | GCCAAACTGCTCTACTCCCGAA |
| *katE*_R |  | CTTCACCCTGGTCAGCGATCTT |
|  |  |  |
|  |  |  |
| *gadA*_F |  | GAAGAATATCCGCAATCCGCAG |
| *gadA*_R |  | GAGCATACAGGCCTCGGAAGAA |
|  |  |  |
| *cstA*_F |  | TTCCATGCGCTGATCTCTTCTG |
| *cstA*_R |  | TGTTCATGGCAAAATACACGCC |
|  |  |  |
|  |  |  |
| *otsA*_F |  | TGCCGACATATGACACCTTGCT |
| *otsA*_R |  | TCAATGCCGATCGGGTAGACTT |
|  |  |  |
| *malE*_F |  | CGAAACAGCGATGACCATCAAC |
| *malE*_R |  | CCGCTTCCAGACCTTCATCAGT |
|  |  |  |
| *ahpC*_F |  | GCAGGGTATCATCCAGGCAATC |
| *ahpC*_R |  | GAGACGGAGCCAGAGTTGCTTC |
|  |  |  |
| *ahpF*_F |  | GATCCCAGCAGCAGTTGAAGGT |
| *ahpF*_R |  | ACGCCTTTGGTGCGATACTGAT |
|  |  |  |
| *rrsG*_F |  | TCAAGGGCACAACCTCCAAGTC |
| *rrsG*_R |  | GGTGTAGCGGTGAAATGCGTAG |

^a^ - Primers are designed by Primer3 software ([www.simgene.com/Primer3](http://www.simgene.com/Primer3))
