# Supplementary material for: Enhancing E. coli Tolerance towards Oxidative Stress via Engineering Its Global Regulator cAMP Receptor Protein (CRP)
Source: PLoS One. 2012 Dec 14;7(12):e51179. doi: 10.1371/journal.pone.0051179 (PMC3522674; doi:10.1371/journal.pone.0051179)
Supplement: Table S4 — DNA microarray and qRT-PCR data comparison of ten selected genes in OM3. (DOC) [file pone.0051179.s011.doc]

**TABLE S4** DNA microarray and qRT-PCR data comparison of ten selected genes in OM3

| Gene | Log_2_ Fold Change in DNA microarray | | Log_2_ Fold Change in qRT-PCR | |
| --- | --- | --- | --- | --- |
|  | |  | |
|  |  | |  | |
|  | Without Stress | Under Stress | Without Stress | Under Stress |
| *sodA* | -0.115 | 0.719 | -0.176 | 0.185 |
| *katE* | 2.701 | 3.801 | 3.132 | 3.048 |
| *ahpC* | -0.518 | 0.667 | -0.302 | -0.031 |
| *ahpF* | -1.051 | -0.374 | -0.893 | -0.483 |
| *gadA* | 4.517 | 7.766 | 5.023 | 7.646 |
| *cstA* | -3.757 | -5.117 | -1.903 | -5.441 |
| *otsA* | 2.580 | 2.996 | 3.199 | 2.906 |
| *malE* | -8.930 | -4.257 | -9.587 | -4.192 |
|  |  |  |  |  |
| *crp* | 4.189 | 3.890 | 3.362 | 2.634 |
|  |  |  |  |  |
| *cya* | 2.240 | 1.263 | 0.215 | 0.755 |
